# A novel immune-related gene pair prognostic signature for predicting overall survival in bladder cancer

**DOI:** 10.1186/s12885-021-08486-0

**Published:** 2021-07-15

**Authors:** Yang Fu, Shanshan Sun, Jianbin Bi, Chuize Kong, Lei Yin

**Affiliations:** 1 Department of Urology, The First Hospital of China Medical University, No. 155 Nanjing North Street, Heping District, Shenyang, 110001 Liaoning Province China; 2 Department of Pharmacy, The First Hospital of China Medical University, Shenyang, Liaoning China

**Keywords:** Immune-related gene pairs, Bladder cancer, Prognosis, Immune cell infiltration, Immune checkpoints, Tumor microenvironment (TME), Tumor mutation burden (TMB)

## Abstract

**Background:**

Bladder cancer (BC) is the ninth most common malignant tumor. We constructed a risk signature using immune-related gene pairs (IRGPs) to predict the prognosis of BC patients.

**Methods:**

The mRNA transcriptome, simple nucleotide variation and clinical data of BC patients were downloaded from The Cancer Genome Atlas (TCGA) database (TCGA-BLCA). The mRNA transcriptome and clinical data were also extracted from Gene Expression Omnibus (GEO) datasets (GSE31684). A risk signature was built based on the IRGPs. The ability of the signature to predict prognosis was analyzed with survival curves and Cox regression. The relationships between immunological parameters [immune cell infiltration, immune checkpoints, tumor microenvironment (TME) and tumor mutation burden (TMB)] and the risk score were investigated. Finally, gene set enrichment analysis (GSEA) was used to explore molecular mechanisms underlying the risk score.

**Results:**

The risk signature utilized 30 selected IRGPs. The prognosis of the high-risk group was significantly worse than that of the low-risk group. We used the GSE31684 dataset to validate the signature. Close relationships were found between the risk score and immunological parameters. Finally, GSEA showed that gene sets related to the extracellular matrix (ECM), stromal cells and epithelial-mesenchymal transition (EMT) were enriched in the high-risk group. In the low-risk group, we found a number of immune-related pathways in the enriched pathways and biofunctions.

**Conclusions:**

We used a new tool, IRGPs, to build a risk signature to predict the prognosis of BC. By evaluating immune parameters and molecular mechanisms, we gained a better understanding of the mechanisms underlying the risk signature. This signature can also be used as a tool to predict the effect of immunotherapy in patients with BC.

**Supplementary Information:**

The online version contains supplementary material available at 10.1186/s12885-021-08486-0.

## Background

There were an estimated 80,470 new cases and 17,670 deaths as a result of bladder cancer (BC) in 2019, and BC is the ninth most common malignant tumor [1]. Nonmuscle-invasive bladder cancer (NMIBC) accounts for 75% of BCs, and 50% of NMIBC cases progress to muscle-invasive bladder cancer (MIBC) [2]. The main treatment for NMIBC is transurethral resection of bladder tumor (TURBT) followed by bladder irrigation, and the treatment strategy for MIBC is usually radical cystectomy combined with cisplatin chemotherapy [3]. The prognosis of patients with BC confined to the mucosa or submucosa is relatively good, and the 5-year survival rate is approximately 80%; however, the 5-year survival rate of BC patients with advanced metastasis is only 15%, and routine treatment has unsatisfactory effects [4, 5]. Therefore, it is essential to identify biomarkers that can reliably predict the prognosis of BC patients and to develop more effective targeted drugs to guide the treatment of BC.

An increasing number of studies have indicated that immune system disorders are closely related to tumorigenesis and development [6–8]. Therefore, immunotherapy has become a promising antitumor strategy in which the body’s own immune response is induced to recognize tumors as foreign antigens and inhibit the proliferation and metastasis of tumor cells by inducing active or passive immune effects [9, 10]. In the past few years, immunotherapy has changed the treatment of solid tumors, and numerous cancer patients have experienced durable responses and long-term survival benefits [11]. To date, Bacillus Calmette-Guerin (BCG) immunotherapy, the gold standard for high-risk NMIBC, has been the most successful; it induces inflammatory cell infiltration and cytokine production in the bladder mucosa, resulting in an immune response against tumor cells [12, 13]. For NMIBC patients with BCG failure, quadruple immunotherapy with BCG, interferon, interleukin-2 (IL-2) and granulocyte-macrophage colony-stimulating factor (GMCSF) has also demonstrated success [14]. However, side effects are very common with BCG, and more than 90% of patients have symptoms of cystitis [15, 16]. In addition, the in-depth study of immune checkpoint inhibitors (ICIs), such as inhibitors of programmed death-1 (PD-1), programmed death ligand-1 (PD-L1) and cytotoxic T lymphocyte antigen-4 (CTLA-4), has led to breakthroughs in immunotherapy [17]. In phase II clinical trials, neoadjuvant use of ICIs in patients with MIBC has shown pathological complete responses [18]. In summary, immunotherapy still has considerable potential in BC. In addition, tumor mutation burden (TMB), also defined as the total number of somatic coding errors, has been considered closely related to tumors [19]. Recent studies also confirmed that TMB was an essential biomarker to predict the effect of ICIs and immunotherapy in tumors, and the TMB level was significantly increased in responders [20–22].

**Fig. 1:**
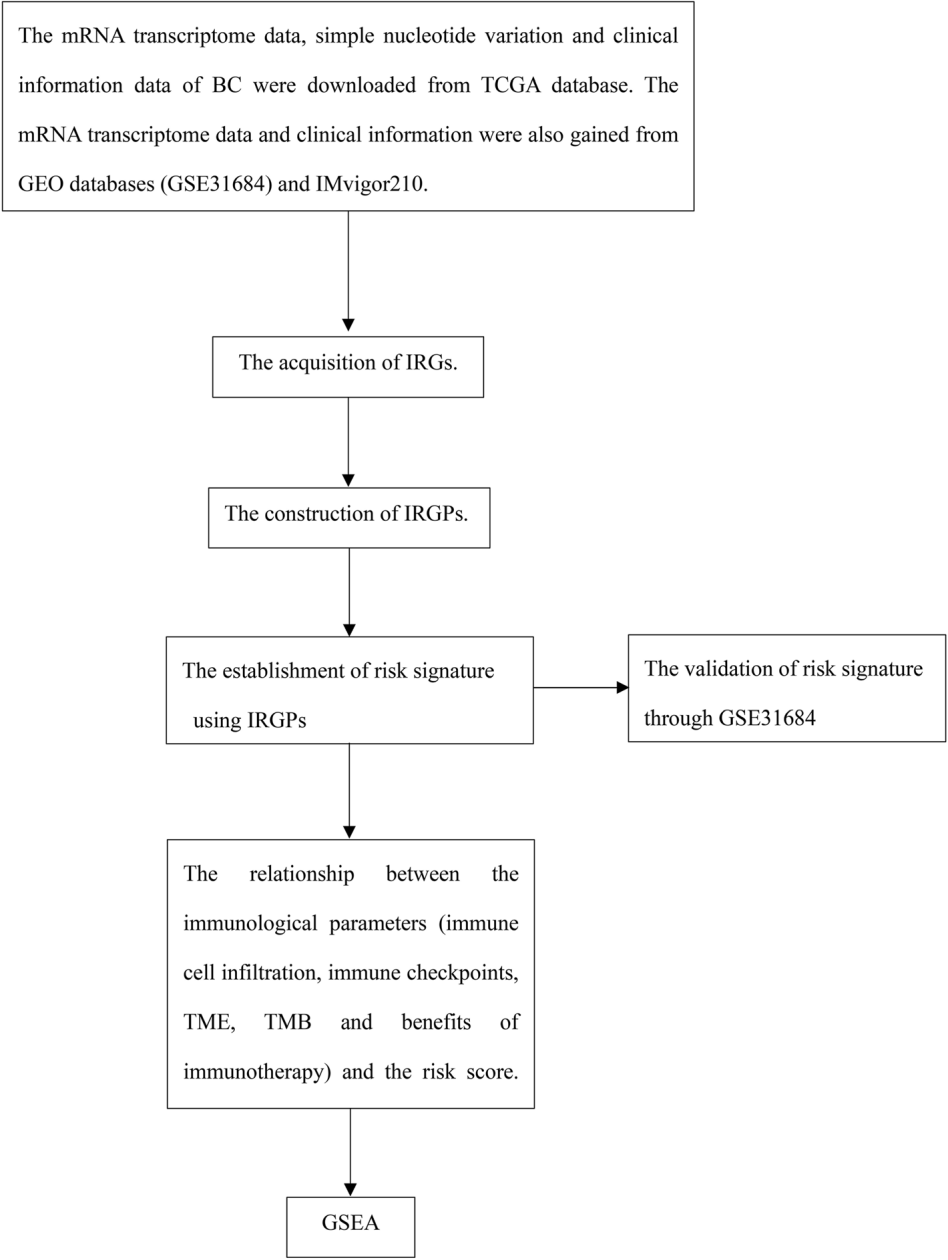
Flow diagram of the current study. BC, bladder cancer; TCGA, The Cancer Genome Atlas; GEO, Gene Expression Omnibus; IRGs, immune-related genes; IRGPs, immune-related gene pairs; TME, tumor microenvironment; TMB, tumor mutation burden; GSEA, gene set enrichment analysis

In this study, we identified immune-related gene pairs (IRGPs) based on immune gene data downloaded from The Cancer Genome Atlas (TCGA) database. The IRGPs related to prognosis were selected to build a risk signature via least absolute shrinkage and selection operator (LASSO) Cox regression. A microarray dataset (GSE31684) obtained from the Gene Expression Omnibus (GEO) database was used to validate the accuracy of the risk signature (Fig. 1). The relationship between the immunological parameters (immune cell infiltration, immune checkpoints, tumor microenvironment (TME) and TMB) and the risk score was investigated. Finally, gene set enrichment analysis (GSEA) was used to explore the molecular mechanisms of the risk score.

## Methods

### Data acquisition

The mRNA transcriptome data, simple nucleotide variation and clinical information of patients with BC were downloaded from the TCGA database (TCGA-BLCA) (https://portal.gdc.cancer.gov/). The mRNA transcriptome data and clinical information were also obtained from GSE31684 (https://www.ncbi.nlm.nih.gov/geo/). After excluding normal samples, 411 patient samples in the TCGA database were analyzed to build a risk signature for evaluating prognosis, and 93 patient samples in GSE31684 were used to validate the signature. A list of immune-related genes (IRGs) was obtained from the Immunology Database and Analysis Portal (ImmPort) [23]. The clinical information of BC patients in the TCGA and GSE31684 databases is shown in Table 1. Then, the TMB of each sample could be calculated as the number of somatic mutations counted in the total length of exons [24]. Moreover, an independent cohort of patients with metastatic urothelial cancer (mUC) receiving PD-1 blockade therapy, as described in the IMvigor210 (mUC) trial, was also included to validate our signature (the mRNA and clinical data were obtained from the package “IMvigor” of R software). The clinical information of BC patients in the TCGA and GSE31684 databases is shown in Table 2. The need for ethical approval was waived because the data we used were obtained from public databases.

**Table 1:** Characteristics of the patients obtained from the TCGA database and GSE31684

Basic information		TCGA (*n* = 409)	GSE31684 (*n* = 72)
Age		69 (median)	68.015 (median)
Gender	Female	106	55
	Male	303	17
Grade	High	385	69
	Low	21	3
	Unknow	3	–
Stage	I & II	132	14
	III & IV	275	58
	Unknow	2	–
T classification	T1 & T2	124	22
	T3 & T4	253	50
	TX	1	–
	Unknow	31	–
N classification	N0	237	44
	N1 &N2 & N3	131	28
	NX	36	–
	Unknow	5	–
M classification	M0	194	41
	M1	11	31
	MX	202	–
	Unknow	2	–

*BC* bladder cancer, *TCGA* the The Cancer Genome Atlas

**Table 2:** Characteristics of the patients obtained from the IMvigor210

Basic information		IMvigor210 (*n* = 348)
Age	Less than 1 year	176
	1–2 years	99
	More than 2 years	73
Gender	Female	76
	Male	272
Subtype	I & II	213
	III & IV	135
Response	CR/PR	68
	SD/PD	230
	NA	50

*BC* bladder cancer, *CR* complete response, *PR* partial response, *SD* stable disease, *PD* progressive disease

### IRGPs

We paired the IRGs in each sample and compared the expression between the two. If the expression of the first IRG was higher than the expression of the second IRG, the value of the IRGP was 1; otherwise, the value was 0 [25]. Then, the gene pairs whose ratio was 0 to 1 in less than 20% of samples were deleted to retain gene pairs that might be related to survival [26].

### The risk signature

These IRGPs and the survival time were considered for further analysis. IRGPs significantly related to prognosis were identified via univariate Cox regression (*P* < 0.05). The risk signature was constructed via LASSO regression, and the number of variables included was reduced and overfitting was effectively avoided by constructing a penalty function. The penalty parameter (λ), a hyperparameter for the risk signature, was determined by tenfold cross-validation following the lowest partial likelihood deviance. The IRGPs included in the risk signature and the corresponding coefficients were obtained through the determined λ value. The risk score was calculated based on the coefficients. The appropriate cutoff value for dividing BC patients into a high-risk group and a low-risk group was determined via the TCGA database by receiver operating characteristic (ROC) curve analysis. The accuracy of the risk signature was also estimated via ROC curve analysis. Kaplan-Meier survival curves and the log-rank test were used to compare the overall survival (OS) between the high-risk group and the low-risk group. Then, univariate and multivariate Cox regression were performed to evaluate whether the risk score was an independent predictor of poor OS in BC patients. Subgroup analyses were performed to prove the robustness of the signature. The GSE31684 and IMvigor210 cohorts were used to validate the signature. In addition, the immune-related signatures from six other articles were compared with the current IRGP signature [27–32].

### Immune parameters

To assess immune infiltration in different risk groups, cell type identification by estimating relative subsets of RNA transcripts (CIBERSORT) was used. CIBERSORT is a deconvolution algorithm that can predict the abundance of 22 immune cells, including naïve B cells, memory B cells, plasma cells, CD8 T cells, naïve CD4 T cells, and resting CD4 memory T cells, based on gene expression profiles (GEPs) [33–35]. The GEPs from the TCGA database were uploaded and used to analyze immune cell infiltration.

The relationship between the risk score and the expression of common immune checkpoints in BC was estimated, including PD-1, PD-L1, CTLA4, lymphocyte activating 3 (LAG3), B and T lymphocyte associated (BTLA) and hepatitis A virus cellular receptor 2 (HAVCR2).

The immune score (the infiltration level of immune cells), the stromal score (the infiltration of stromal cells) and tumor purity were calculated using the GEPs of TCGA database via Estimation of Stromal and Immune cells in Malignant Tumor tissues using Expression data (ESTIMATE) to explore the TME further [36, 37]. Then, we investigated the relationship between the risk score and the results of ESTIMATE. The impacts of the immune score and stromal score on the survival of all BC patients were also evaluated via the Kaplan–Meier method. Finally, we assessed whether there was a correlation between the risk score and the TMB.

The GSE31684 and publicly available “IMvigor210” datasets were used to perform the same analysis to determine the changes in immune parameters and risk score.

### Gene set enrichment analysis (GSEA)

In the current study, we used GSEA to explore the molecular mechanisms underlying the risk score. The gene sets of “c2.cp.kegg.v7.1.symbols.gmt” and “c5.go.v7.2.symbols.gmt” from the Molecular Signatures Database (MSigDB) were downloaded for further analysis. The phenotype labels were high-risk group and low-risk group. Normalized enrichment scores (NESs), the nominal *P* value (NOM *P* value) and the false discovery rate Q value (FDR Q value) were acquired. NOM *P* value < 0.05 and FDR Q value < 0.25 were considered to indicate significant enrichment.

### Statistical analysis

All statistical analyses were performed using R 4.03 software. The log-rank test and univariate and multivariate Cox regression were completed via the survival package of R. LASSO Cox regression was performed via the glmnet package of R. All ROC curves were generated via the survival ROC package of R. The nomogram and the calibration were illustrated via the rms package of R. ESTIMATE analysis was achieved via the estimate package of R. Fisher’s exact tests were used to estimate differences in clinical variables between the groups in the TCGA database. Pearson test was used for all correlation analysis.

## Results

### Construction of the IRGP signature

**Fig. 2:**
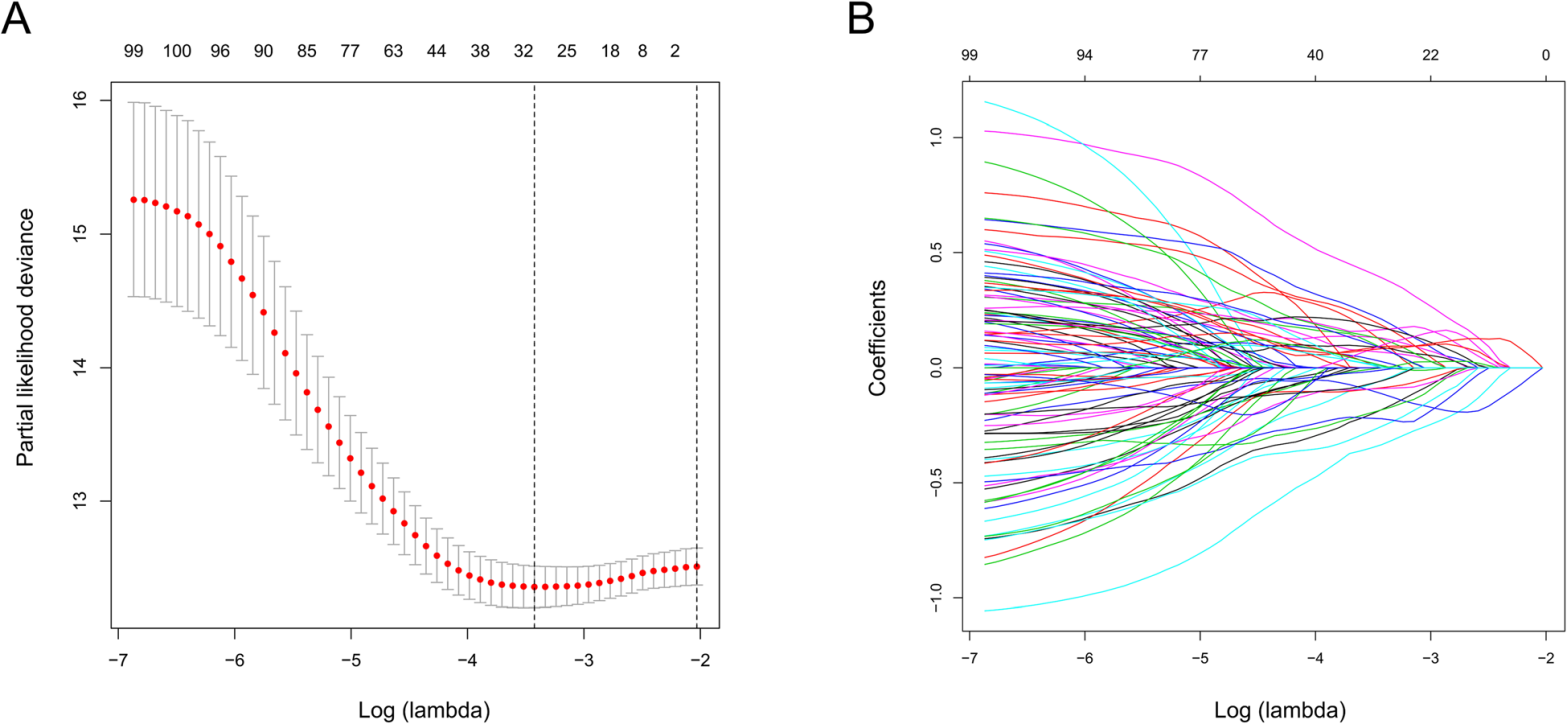
Construction of the IRGP signature by LASSO regression analysis. LASSO coefficient profiles of the included genes in TCGA-BLCA (**A**). Selection of the optimal parameter (λ) in the LASSO model for TCGA-BLCA (**B**). LASSO, least absolute shrinkage and selection operator; BLCA, bladder cancer; IRGP, immune-related gene pair

**Fig. 3:**
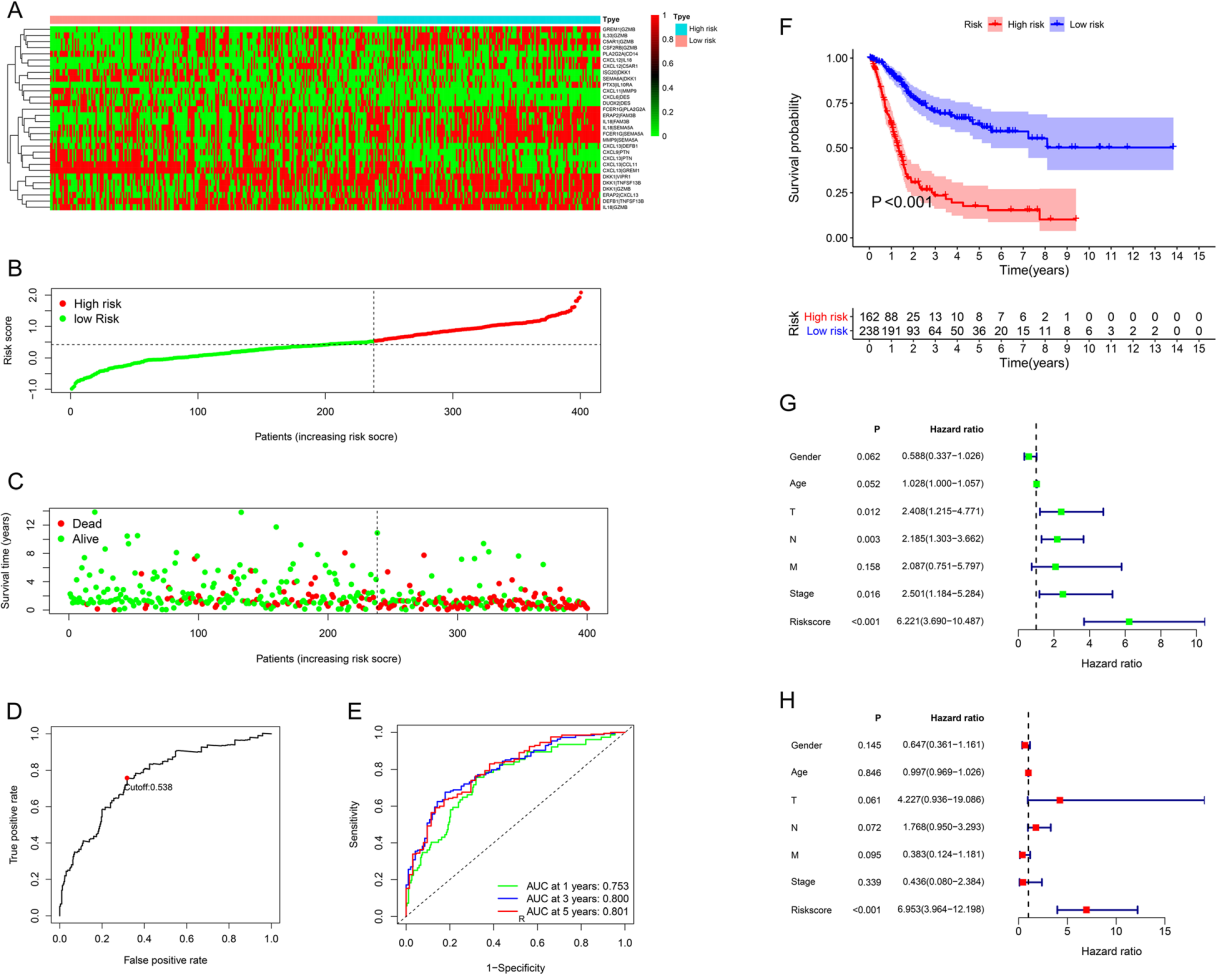
Characteristics of the IRGP signature using the TCGA. The score of included IRGPs in different groups (**A**). The categorization of BC patients into different groups (**B**). The survival status of patients in the high-risk group and low-risk group (**C**). Through ROC curve analysis, the cutoff value for dividing BC patients into the high-risk group and the low-risk group was determined to be 0.538 (**D**). The AUC of the ROC curve results showed a moderate prognostic power of the risk score (**E**). The results of Kaplan-Meier survival analysis revealed that a high-risk score was significantly related to poor OS (**F**). Univariate and multivariate Cox regression analyses demonstrated that the risk score was an independent prognostic factor (**G**-**H**). TCGA, The Cancer Genome Atlas; LASSO, least absolute shrinkage and selection operator; BLCA, bladder cancer; BC, bladder cancer; IRGPs, immune-related gene pairs; AUC, area under curve; ROC, receiver operating characteristic; OS, overall survival

**Fig. 4:**
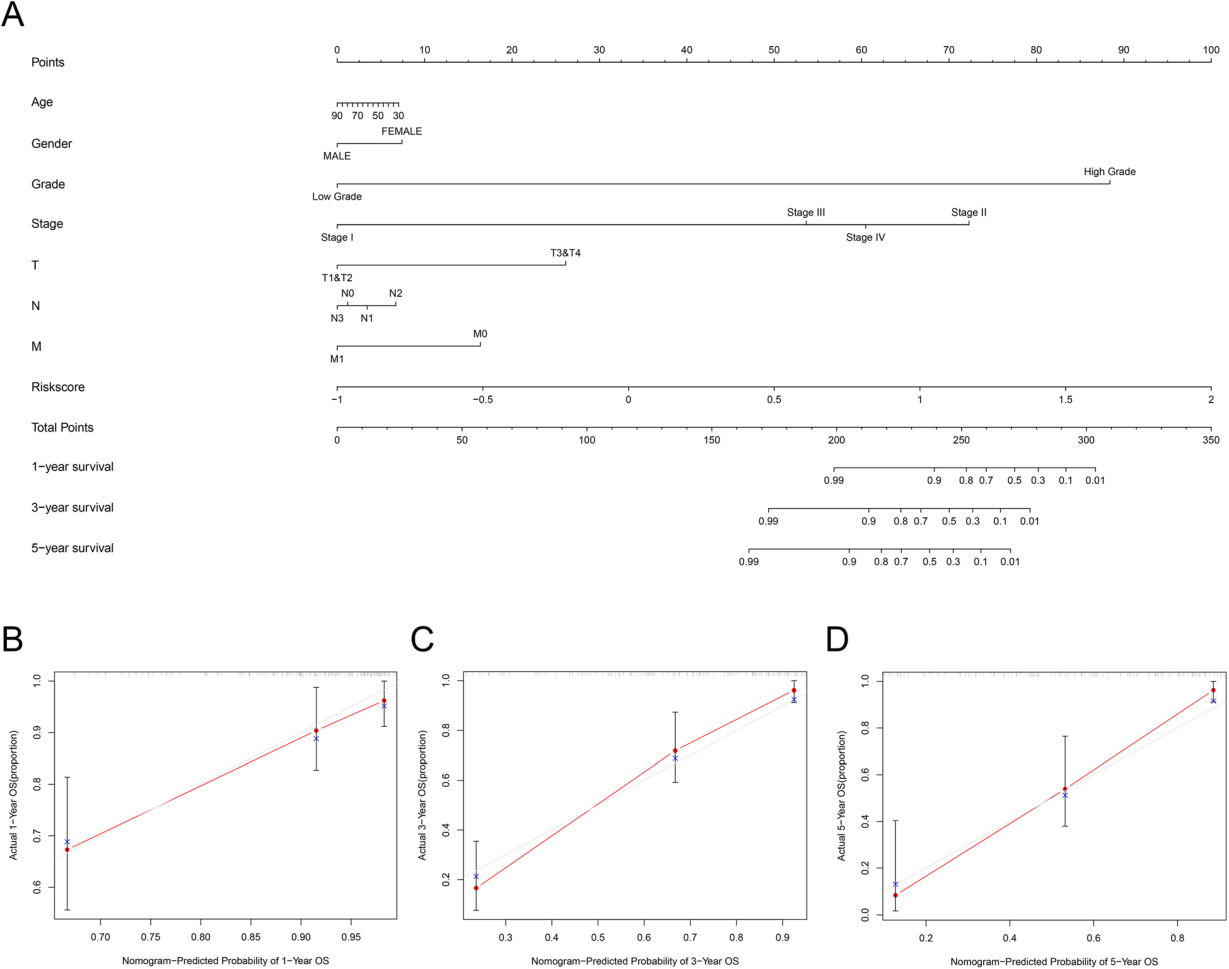
Nomogram (TCGA). The nomogram could predict the probability of 1-year, 3-year and 5-year OS (**A**). The calibration curve revealed the accuracy of the nomogram for predicting 1-year (**B**), 3-year (**C**) and 5-year (**D**) OS. TCGA, The Cancer Genome Atlas; OS, overall survival

**Fig. 5:**
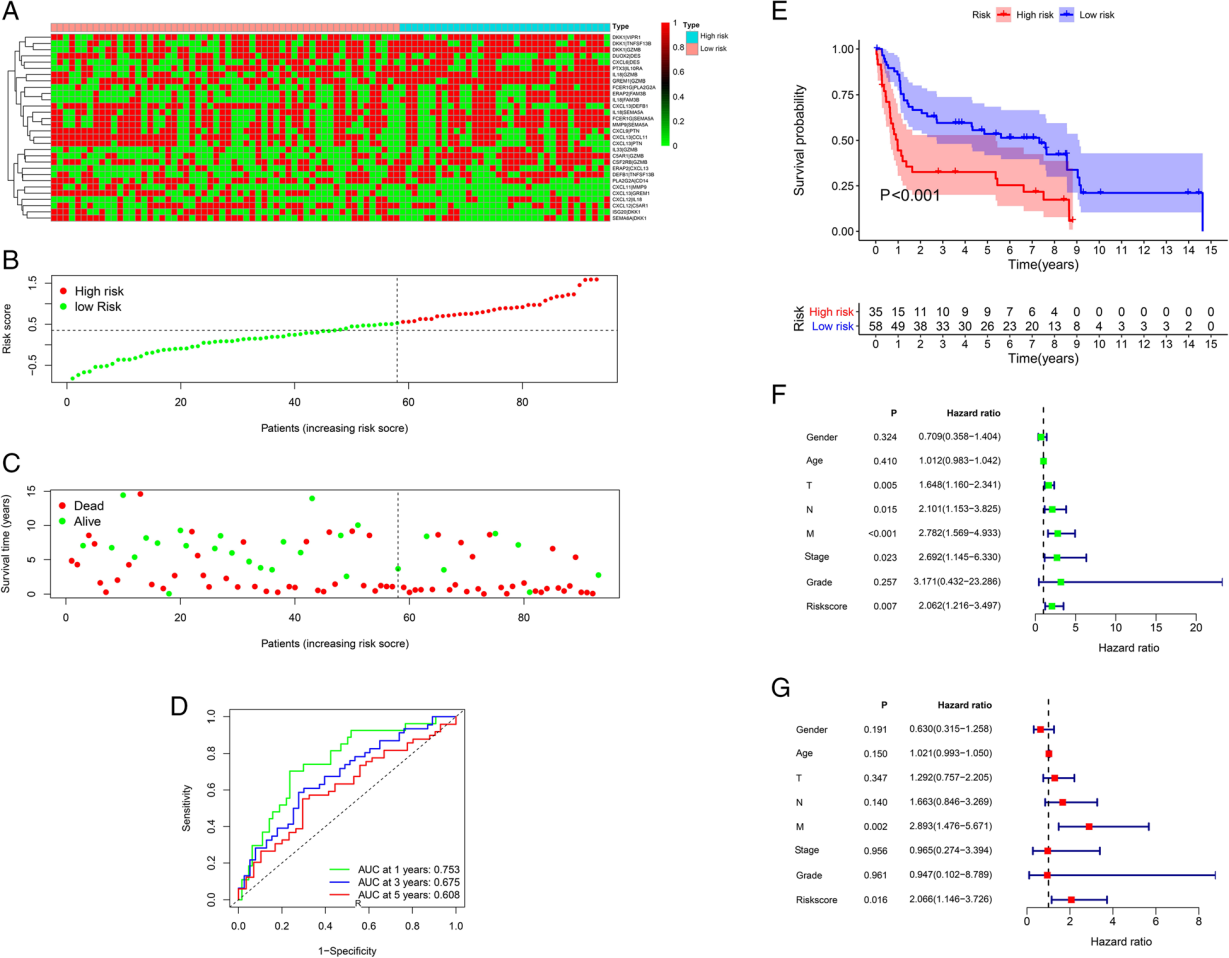
Validation of the IRGP signature using GSE31684. The score of included IRGPs in different groups (**A**). The categorization of BC patients into different groups (**B**). The survival status of patients in the high-risk group and low-risk group (**C**). The AUC of the ROC curve results showed a moderate prognostic power of the risk score (**D**). The results of Kaplan-Meier survival analysis revealed that a high-risk score was significantly related to poor OS (**E**). Univariate and multivariate Cox regression analyses demonstrated that the risk score was an independent prognostic factor (**F**-**G**). BC, bladder cancer; IRGPs, immune-related gene pairs; AUC, area under the curve; ROC, receiver operating characteristic; OS, overall survival

**Fig. 6:**
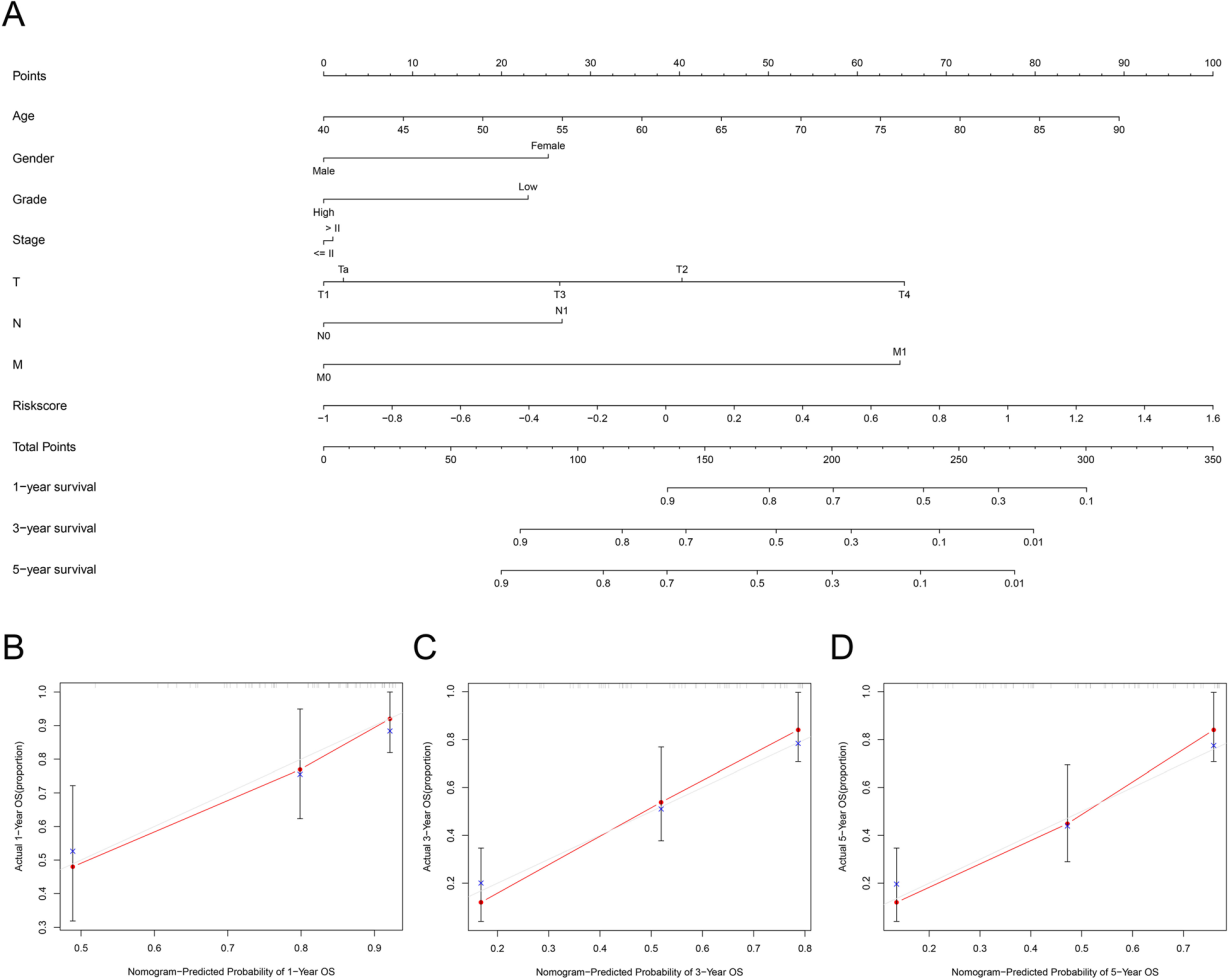
Nomogram (GEO). The nomogram could predict the survival probability of 1-year, 3-year and 5-year OS (**A**). The calibration curve revealed the accuracy of the nomogram for predicting 1-year (**B**), 3-year (**C**) and 5-year (**D**) OS. GEO, Gene Expression Omnibus; OS, overall survival

**Fig. 7:**
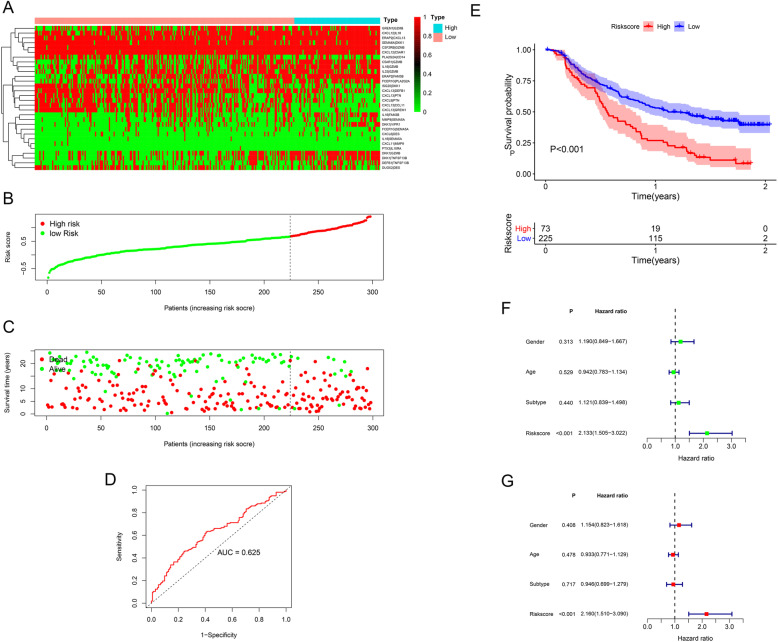
Validation of the IRGP signature using IMvigor210. The scores of the included IRGPs in different groups (**A**). The categorization of BC patients into different groups (**B**). The survival status of patients in the high-risk group and low-risk group (**C**). The AUC of the ROC curve results showed that the risk score had moderate prognostic power (**D**). Kaplan-Meier survival analysis revealed that a high risk score was significantly related to poor OS (**E**). Univariate and multivariate Cox regression analyses demonstrated that the risk score was an independent prognostic factor (**F**-**G**). BC, bladder cancer; IRGPs, immune-related gene pairs; AUC, area under the curve; ROC, receiver operating characteristic; OS, overall survival

**Fig. 8:**
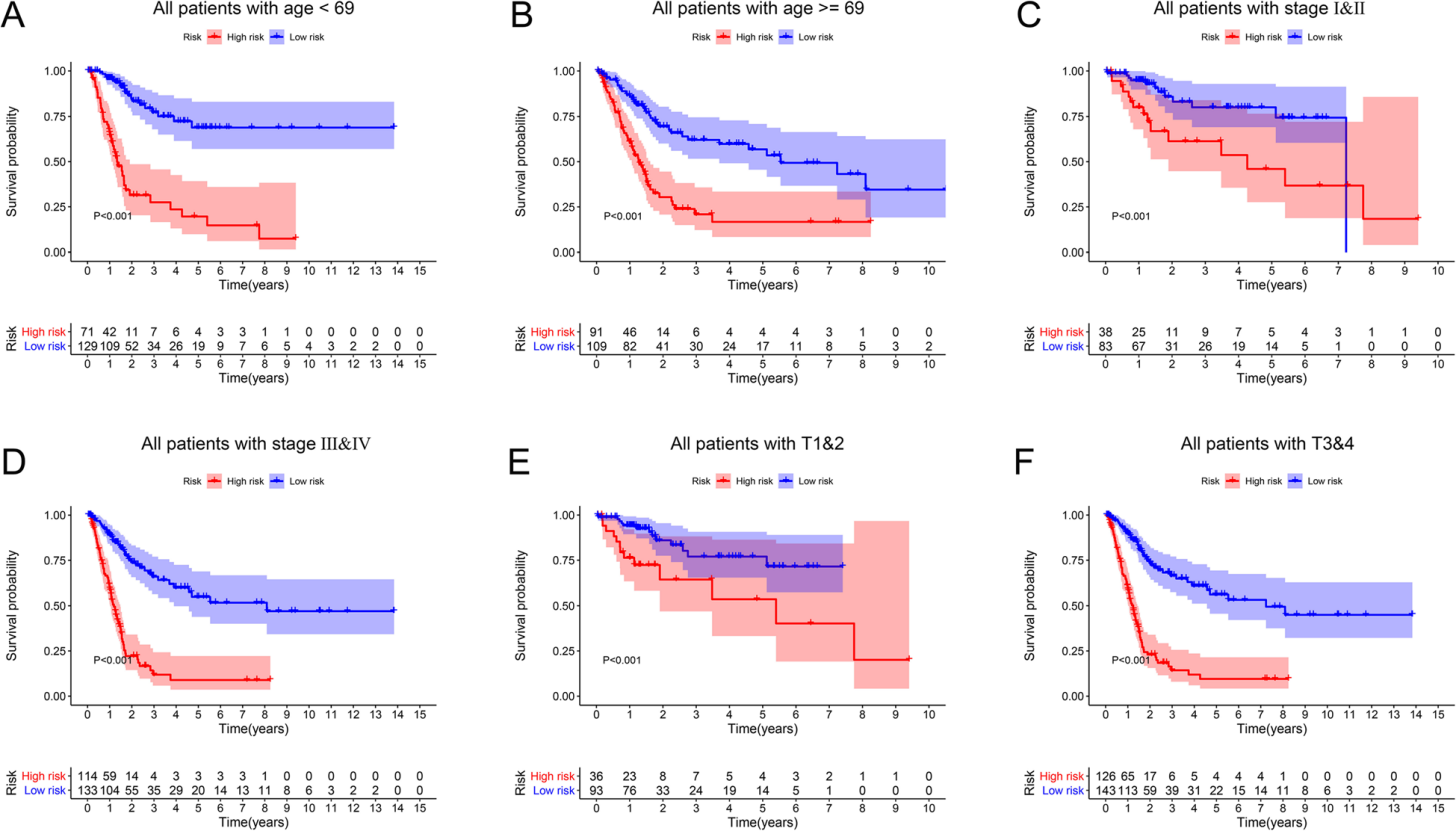
Subgroup analyses (TCGA). Subgroup analyses were performed based on age (**A**-**B**), clinical stage (**C**-**D**) and T stage (**E**-**F**) to confirm the robustness of the risk signature. TCGA, The Cancer Genome Atlas

After filtering out unqualified IRGPs, 652 IRGPs remained. Then, 104 IRGPs significantly related to prognosis were identified via univariate Cox regression (*P* < 0.05) and used as candidate pairs for signature building. The risk score was calculated based on the coefficients of the selected IRGPs obtained from LASSO Cox regression (the optimal λ was 0.03253915) (Fig. 2A-B). Ultimately, 30 IRGPs were included in the risk signature (Table 3, Fig. 3A-C). Through ROC curve analysis, the cutoff value that divided BC patients into the high-risk group and low-risk group was determined to be 0.538 (Fig. 3D). The ROC curve results showed a moderate prognostic power of the risk score [area under the curve (AUC) at 1 year = 0.758, AUC at 3 years = 0.800, AUC at 5 years = 0.801] (Fig. 3E). The results of Kaplan-Meier survival analyses and Fisher’s exact tests revealed that a high risk score was significantly correlated with advanced age (*P* = 0.021), advanced clinical stage (*P* < 0.001), high T classification (*P* = 0.007), high N classification (*P* = 0.002), high M classification (*P* < 0.001) and poor OS (*P* < 0.001) (Table 4, Fig. 3F). Univariate Cox regression showed that T stage [hazard ratio (HR) = 2.408, 95% confidence interval (CI) = 1.215–4.771, *P* = 0.012], N stage (HR = 2.185, 95% CI = 1.303–3.662, *P* = 0.003), clinical stage (HR = 2.501, 95% CI = 1.184–5.284, *P* = 0.016) and risk score (HR = 6.221, 95% CI = 3.690–10.487, *P* < 0.001) were closely related to poor prognosis in BC (Fig. 3G). We then performed multivariate Cox regression, which identified only the risk score as associated with prognosis (HR = 6.953, 95% CI = 3.964–12.198, *P* < 0.001) (Fig. 3H). The nomogram could predict the survival probability of 1-year, 3-year and 5-year OS (Fig. 4A). The calibration curve revealed the accuracy of the prediction using the nomogram (Fig. 4B-D). To validate that the risk signature performed similarly for other datasets, the independent cohort from the GSE31684 dataset was employed for external validation. We divided BC patients from GSE31684 into a high-risk group and a low-risk group (Fig. 5A-C). The ROC curve results showed a moderate prognostic power for BC patients of the risk score (AUC at 1 year = 0.753, AUC at 3 years = 0.675, AUC at 5 years = 0.608) (Fig. 5D). Kaplan-Meier curves revealed that the prognosis of the high-risk group was worse than that of the low-risk group (*P* < 0.001) (Fig. 5E). The Cox regression results indicated that the risk score was an independent predictor of poor OS in BC (Fig. 5F-G). The nomogram could predict the survival probability of 1-year, 3-year and 5-year OS (Fig. 6A). The calibration curve revealed the accuracy of the prediction using the nomogram (Fig. 6B-D). Another cohort from the IMvigor210 dataset was also subjected to the same analysis to validate the signature (Fig. 7A-G). A series of subgroup analyses performed on data from the TCGA (Fig. 8A-F), GSE31684 (Supplementary Fig. S1A-F) and IMvigor210 (Supplementary Fig. S2A-D) indicated that the risk signature was robust.

**Table 3:** Information on the 30 selected IRGPs

Gena pair 1	Full name	Gene pair 2	Full name	Coefficient
FCER1G	Fc fragment of IgE receptor Ig	PLA2G2A	phospholipase A2 group IIA	0.115
FCER1G	Fc fragment of IgE receptor Ig	SEMA5A	semaphorin 5A	0.085
ERAP2	endoplasmic reticulum aminopeptidase 2	CXCL13	C-X-C motif chemokine ligand 13	0.025
ERAP2	endoplasmic reticulum aminopeptidase 2	FAM3B	FAM3 metabolism regulating signaling molecule B	0.390
CXCL9	C-X-C motif chemokine ligand 9	PTN	pleiotrophin	−0.182
CXCL11	C-X-C motif chemokine ligand 11	MMP9	matrix metallopeptidase 9	−0.334
CXCL6	C-X-C motif chemokine ligand 6	DES	desmin	−0.055
CXCL12	C-X-C motif chemokine ligand 12	IL18	interleukin 18	0.210
CXCL12	C-X-C motif chemokine ligand 12	C5AR1	complement C5a receptor 1	0.128
CXCL13	C-X-C motif chemokine ligand 13	DEFB1	defensin beta 1	−0.010
CXCL13	C-X-C motif chemokine ligand 13	CCL11	C-C motif chemokine ligand 11	−0.087
CXCL13	C-X-C motif chemokine ligand 13	GREM1	gremlin 1, DAN family BMP antagonist	−0.112
CXCL13	C-X-C motif chemokine ligand 13	PTN	pleiotrophin	−0.254
DEFB1	defensin beta 1	TNFSF13B	TNF superfamily member 13b	0.183
MMP9	matrix metallopeptidase 9	SEMA5A	semaphorin 5A	0.164
ISG20	interferon stimulated exonuclease gene 20	DKK1	dickkopf WNT signaling pathway inhibitor 1	−0.183
DUOX2	dual oxidase 2	DES	desmin	−0.090
PLA2G2A	phospholipase A2 group IIA	CD14	CD14 molecule	−0.221
IL18	interleukin 18	SEMA5A	semaphorin 5A	0.066
IL18	interleukin 18	FAM3B	FAM3 metabolism regulating signaling molecule B	0.087
IL18	interleukin 18	GZMB	granzyme B	0.124
PTX3	pentraxin 3	IL10RA	interleukin 10 receptor subunit alpha	0.173
SEMA6A	semaphorin 6A	DKK1	dickkopf WNT signaling pathway inhibitor 1	−0.057
C5AR1	complement C5a receptor 1	GZMB	granzyme B	0.091
DKK1	dickkopf WNT signaling pathway inhibitor 1	TNFSF13B	TNF superfamily member 13b	0.084
DKK1	dickkopf WNT signaling pathway inhibitor 1	VIPR1	vasoactive intestinal peptide receptor 1	0.007
DKK1	dickkopf WNT signaling pathway inhibitor 1	GZMB	granzyme B	0.097
GREM1	gremlin 1, DAN family BMP antagonist	GZMB	granzyme B	0.082
IL33	interleukin 33	GZMB	granzyme B	0.009
CSF2RB	colony stimulating factor 2 receptor subunit beta	GZMB	granzyme B	< 0.001

**Table 4:** Differences in the characteristics of BC patients between the high risk and low risk in TCGA

Basic information		Low risk	High risk	*P* value
Age				0.021
	≤ 69	138	75	
	> 69	100	87	
Gender				0.143
	Female	55	48	
	Male	183	114	
Stage				< 0.001
	I&II	93	36	
	III&IV	143	126	
T				0.007
	T1&T2	83	38	
	T3&T4	133	114	
N				0.002
	N0	152	81	
	N1–3	61	65	
M				< 0.001
	M0	132	61	
	M1	2	9

**Fig. 9:**
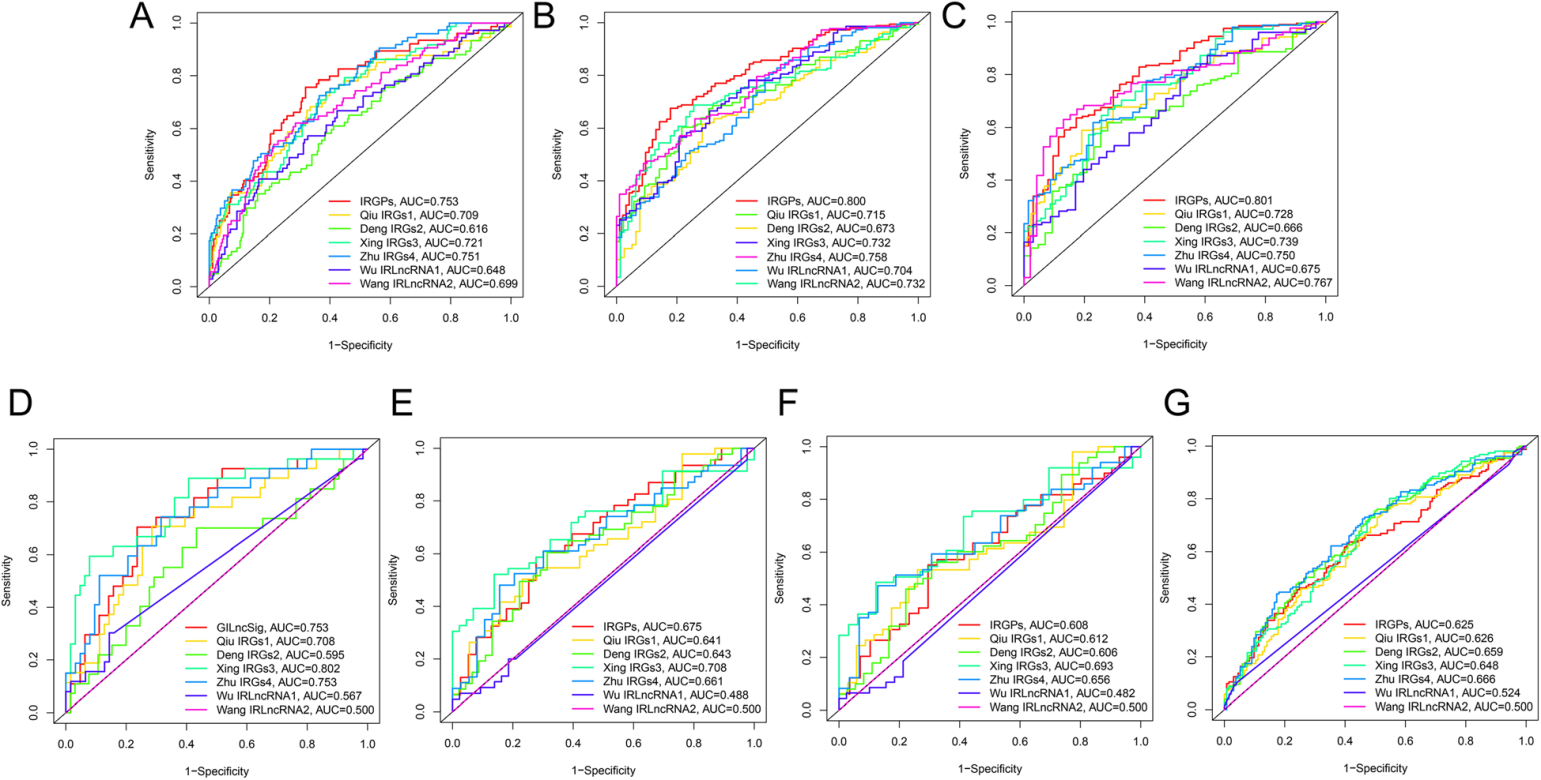
Comparison of signatures. AUC for multiple signatures at 1 year (**A**), 3 years (**B**), and 5 years (**C**) using TCGA datasets via ROC curves. AUC for multiple signatures at 1 year (**D**), 3 years (**E**), and 5 years (**F**) using GEO datasets via ROC curves. AUC for multiple signatures at 1 year (**G**) using IMvigor210 datasets via ROC curves. AUC, area under the curve; ROC, receiver operating characteristic; TCGA, The Cancer Genome Atlas; GEO, Gene Expression Omnibus; IRGPs, immune-related gene pairs; IRGs, immune-related gene; IRLncRNA, immune-related long non-coding RNA

A comparison of the current model with the previous model by using data from the TCGA (Fig. 9A-C), GEO (Fig. 9D-F) and IMvigor210 (Fig. 9G), revealed that the model had acceptable accuracy, with the largest AUC value obtained with data from the TCGA.

### Evaluation of immune parameters

**Fig. 10:**
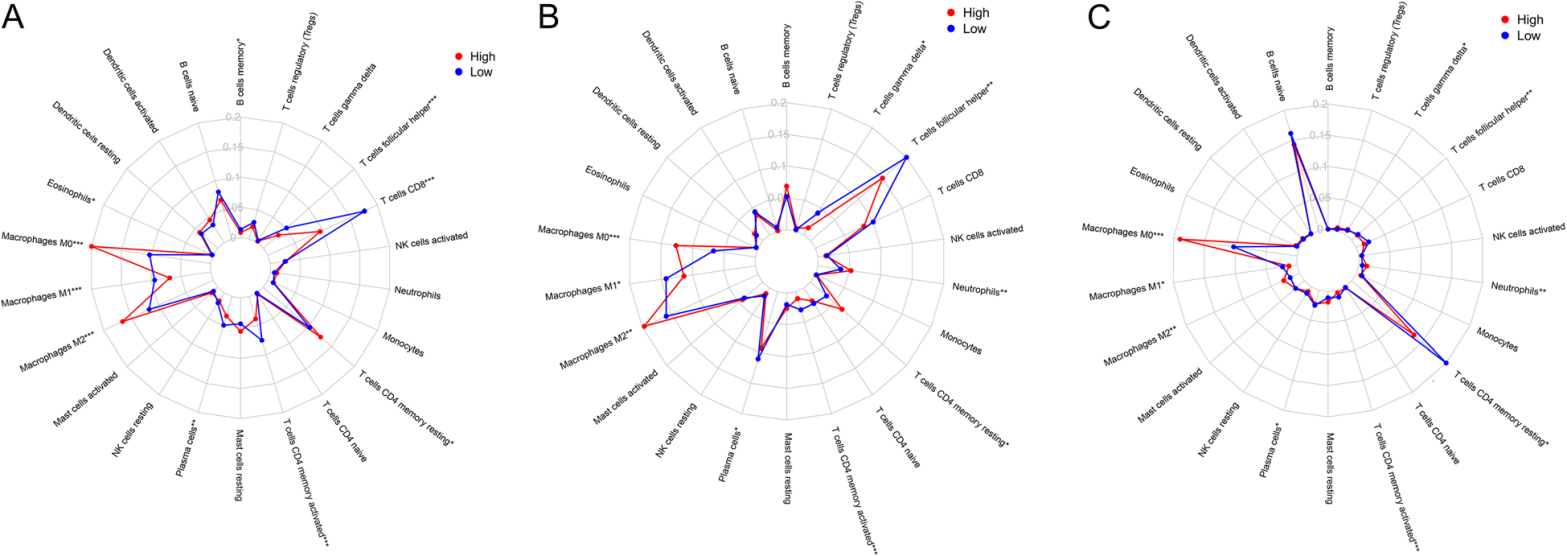
Immune cells and risk scores. In the TCGA, the levels of memory B cells, plasma cells, M1 macrophages, CD8 T cells, activated CD4 memory T cells and follicular helper T cells were higher in the low-risk group than in the high-risk group, and the levels of M0 macrophages, M2 macrophages, eosinophils and resting CD4 memory T cells were higher in the high-risk group than in the low-risk group (**A**). The other two databases, GSE31684 (**B**) and IMvigor210 (**C**), were used to verify the related changes in immune cells in the TCGA database. The results obtained from the three datasets were mostly consistent. TCGA, The Cancer Genome Atlas; *, *P* < 0.05; **, *P* < 0.01; **, *P* < 0.001

CIBERSORT was used to evaluate immune cell infiltration in different risk groups. The results were visualized by radar plot. Memory B cell memory, resting memory CD4 T cells, eosinophils, plasma cells, CD8 T cells, activated memory CD4 T cells, follicular helper T cells, M0 macrophages, M1 macrophages and M2 macrophages were differentially enriched in the different risk groups. The levels of memory B cells (*P* = 0.040), plasma cells (*P* = 0.006), M1 macrophages (*P* < 0.001), CD8 T cells (*P* < 0.001), activated memory CD4 T cells (*P* < 0.001) and follicular helper T cells (*P* < 0.001) were higher in the low-risk group than in the high-risk group. The levels of M0 macrophages (*P* < 0.001), M2 macrophages (*P* < 0.001), eosinophils (*P* = 0.031) and resting memory CD4 T cells (*P* = 0.022) were higher in the high-risk group than in the low-risk group (Fig. 10A). The other two datasets, GSE31684 (Fig. 10B) and IMvigor210 (Fig. 10C), were used to verify the related changes in immune cells in the TCGA database. The results obtained from the three datasets were mostly consistent.

**Fig. 11:**
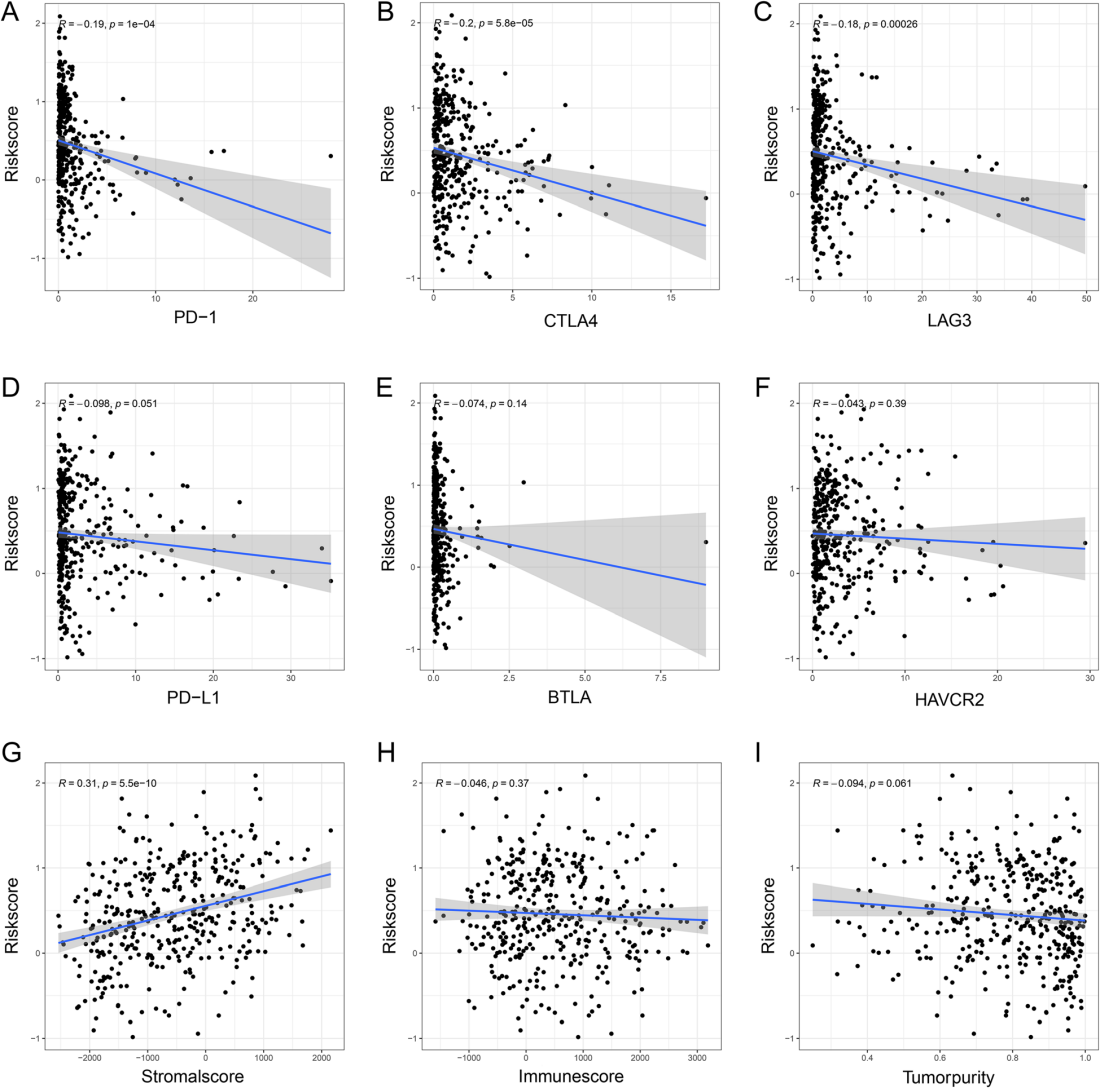
Immunological parameters and risk scores (TCGA). The expression levels of PD-1 (**A**), CTLA4 (**B**), and LAG3 (**C**) were significantly negatively correlated with the risk score. However, there was no significant difference between the risk score and other immune checkpoints, including PD-L1 (**D**), BTLA (**E**), and HAVCR2 (**F**). The stromal score (**G**) was significantly positively correlated with the risk score. There was no significant correlation between the immune score (**H**), tumor purity (**I**) and risk score. TCGA, The Cancer Genome Atlas; PD-1, programmed death-1; CTLA-4, cytotoxic T lymphocyte antigen-4; LAG3, lymphocyte activating 3; PD-L1, programmed death ligand-1; BTLA, B and T lymphocyte associated; HAVCR2, hepatitis A virus cellular receptor 2; BC, bladder cancer

Then, the relationship between the risk score and the expression of common immune checkpoints in BC was explored. We found that the expression levels of PD-1 (correlation coefficient = − 0.19, *P* < 0.001) (Fig. 11A), CTLA4 (correlation coefficient = − 0.20, *P* < 0.001) (Fig. 11B), and LAG3 (correlation coefficient = − 0.18, *P* < 0.001) (Fig. 11C) were significantly negatively correlated with the risk score. However, there was no significant difference between the risk score and other immune checkpoints, including PD-L1 (correlation coefficient = − 0.098, *P* = 0.051) (Fig. 11D), BTLA (correlation coefficient = − 0.74, *P* = 0.140) (Fig. 11E), and HAVCR2 (correlation coefficient = − 0.043, *P* = 0.390) (Fig. 11F). We also confirmed that the stromal score was significantly positively correlated with the risk score (correlation coefficient = 0.31, *P* < 0.001) (Fig. 11G). However, there was no significant correlation between the immune score and tumor purity (correlation coefficient = − 0.046, *P* = 0.370) (Fig. 11H) or risk score (correlation coefficient = − 0.094, *P* = 0.061) (Fig. 11I). The related immune changes were also observed using GSE31684 (Supplementary Fig. S3A-I) and IMvigor210 (Supplementary Fig. S4A-I). The results obtained from the three datasets were mostly consistent.

**Fig. 12:**
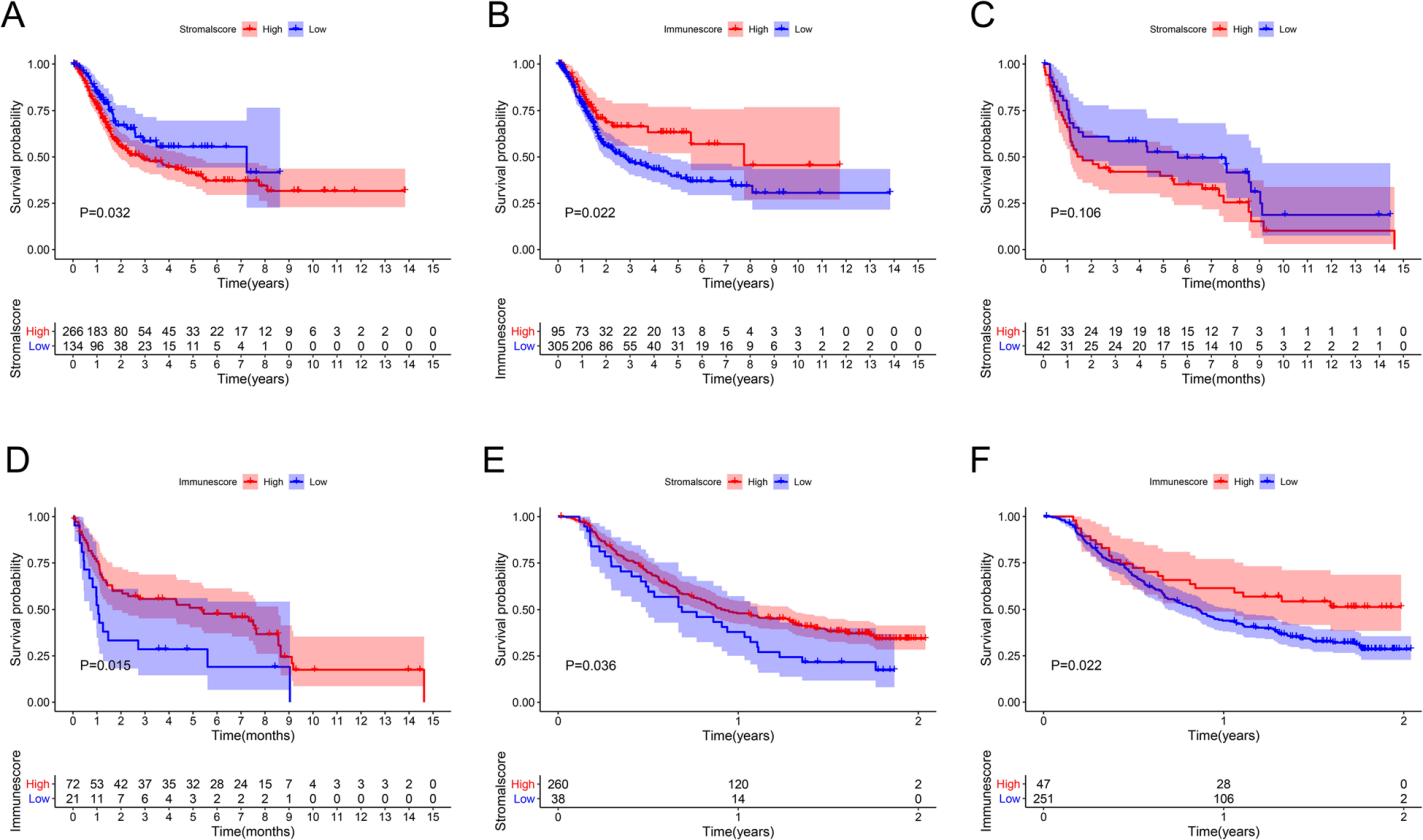
Immune microenvironment and prognosis. A high stromal score (**A**) and a low immune score (**B**) indicated a poor prognosis in the TCGA. We performed the same survival analysis using GSE31684 (**C**-**D**) and IMvigor210 (**E**-**F**). The results obtained from the three datasets revealed that a high immune score was closely related to a good prognosis. TCGA, The Cancer Genome Atlas

A high stromal score (*P* = 0.032) (Fig. 12A) and a low immune score indicated a poor prognosis in BC (*P* = 0.022) (Fig. 12B). We performed the same survival analysis using GSE31684 (Fig. 12C-D) and IMvigor210 (Fig. 12E-F). The results obtained from the three datasets revealed that a high immune score was closely correlated with a good prognosis.

**Fig. 13:**
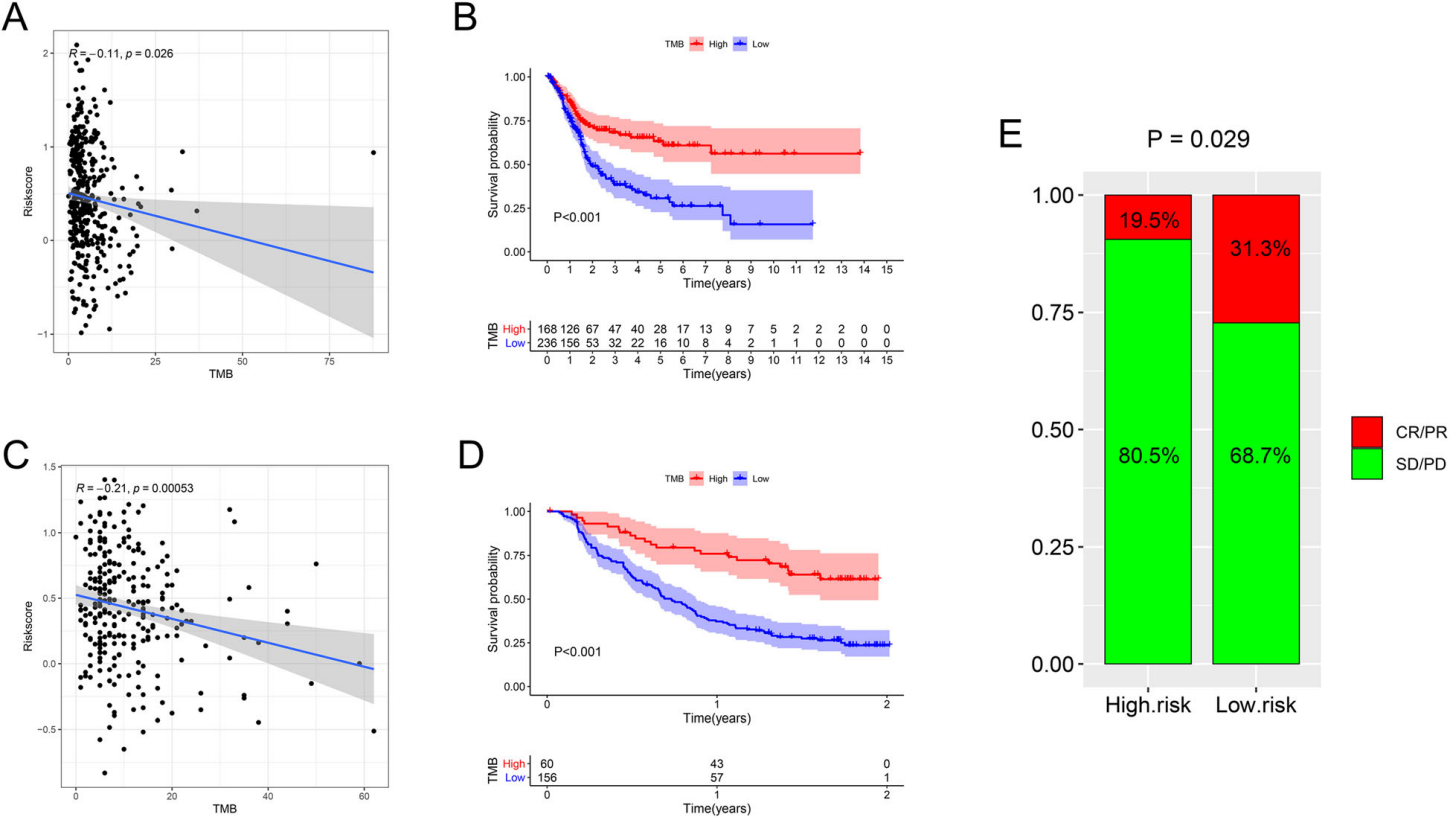
TMB. In the TCGA, the level of TMB was significantly negatively correlated with the risk score (**A**), and an increased level of TMB correlated with improved OS (*P* < 0.001) (**B**). These results were consistent with the results obtained from IMvigor210 (**C**-**D**). Moreover, in the IMvigor210 cohort, low-risk patients had more significant immunotherapy effects (PD-1 blockade therapy) (**E**). TMB, tumor mutation burden; TCGA, The Cancer Genome Atlas; OS, overall survival; CR, complete response, PR, partial response, SD, stable disease; PD, progressive disease

In the TCGA, the level of TMB was significantly negatively correlated with the risk score (correlation coefficient = − 0.11, *P* = 0.026) (Fig. 13A), and an increased level of TMB correlated with improved OS (*P* < 0.001) (Fig. 13B). These results were consistent with the results obtained from IMvigor210 (Fig. 13C-D). Moreover, in the IMvigor210 cohort, low-risk patients had more significant immunotherapy effects (PD-1 blockade therapy) (Fig. 13E).

### GSEA

**Fig. 14:**
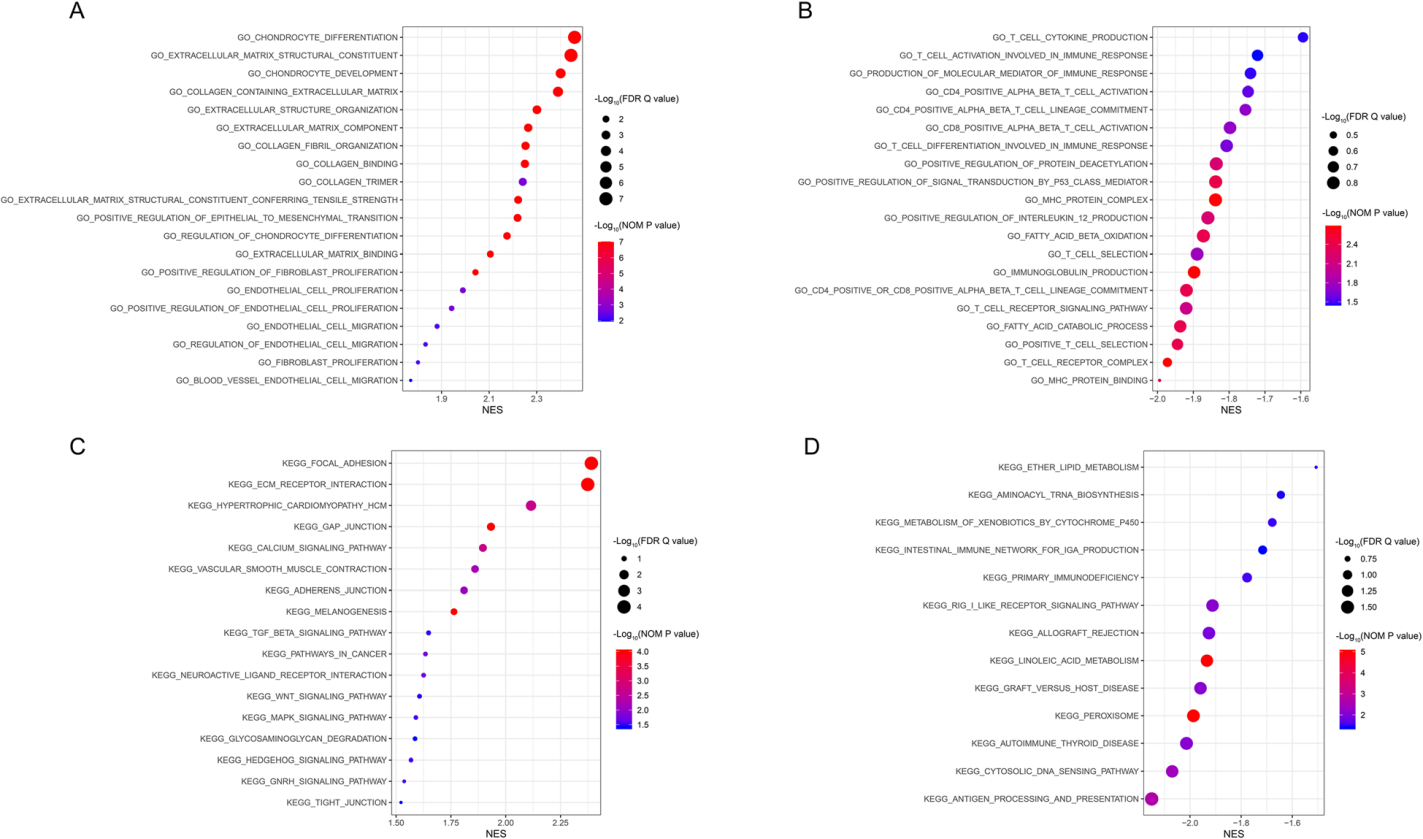
GSEA. Gene sets enriched in the high-risk phenotype via GO (**A**). Gene sets enriched in the low-risk phenotype via GO (**B**). Gene sets enriched in the high-risk phenotype via KEGG (**C**). Gene sets enriched in the low-risk phenotype via KEGG (**D**). GSEA, gene set enrichment analysis; GO, Gene Ontology; KEGG, Kyoto Encyclopedia of Genes and Genomes; NES, normalized enrichment scores; NOM *P* value, nominal *P* value; FDR Q value, false discovery rate Q value

We used GSEA to explore the molecular mechanisms underlying the risk score. The Gene Ontology (GO) results, as shown in Table 5 and Fig. 14A, revealed the most significant signaling pathways enriched in the high-risk phenotype. The GO results, as shown in Table 6 and Fig. 14B, revealed the most significant signaling pathways enriched in the low-risk phenotype. The Kyoto Encyclopedia of Genes and Genomes (KEGG) results, as shown in Table 7 and Fig. 14C, revealed the most significant signaling pathways enriched in the high-risk phenotype. The KEGG results, as shown in Table 8 and Fig. 14D, revealed the most significant signaling pathways enriched in the low-risk phenotype [38].

**Table 5:** Gene sets enriched in the high risk phenotype via GO

Gene set name	NES	NOM *p*-val	FDR *q*-val
GO_CHONDROCYTE_DIFFERENTIATION	2.459	< 0.001	< 0.001
GO_EXTRACELLULAR_MATRIX_STRUCTURAL_CONSTITUENT	2.444	< 0.001	< 0.001
GO_CHONDROCYTE_DEVELOPMENT	2.400	< 0.001	< 0.001
GO_COLLAGEN_CONTAINING_EXTRACELLULAR_MATRIX	2.389	< 0.001	< 0.001
GO_EXTRACELLULAR_STRUCTURE_ORGANIZATION	2.301	< 0.001	0.001
GO_EXTRACELLULAR_MATRIX_COMPONENT	2.264	< 0.001	0.002
GO_COLLAGEN_FIBRIL_ORGANIZATION	2.253	< 0.001	0.002
GO_COLLAGEN_BINDING	2.250	< 0.001	0.002
GO_COLLAGEN_TRIMER	2.241	0.002	0.003
GO_EXTRACELLULAR_MATRIX_STRUCTURAL_CONSTITUENT_CONFERRING_TENSILE_STRENGTH	2.222	< 0.001	0.003
GO_POSITIVE_REGULATION_OF_EPITHELIAL_TO_MESENCHYMAL_TRANSITION	2.219	< 0.001	0.003
GO_REGULATION_OF_CHONDROCYTE_DIFFERENTIATION	2.175	< 0.001	0.005
GO_EXTRACELLULAR_MATRIX_BINDING	2.105	< 0.001	0.011
GO_POSITIVE_REGULATION_OF_FIBROBLAST_PROLIFERATION	2.042	< 0.001	0.018
GO_ENDOTHELIAL_CELL_PROLIFERATION	1.990	0.002	0.024
GO_POSITIVE_REGULATION_OF_ENDOTHELIAL_CELL_PROLIFERATION	1.942	0.002	0.031
GO_ENDOTHELIAL_CELL_MIGRATION	1.881	0.006	0.040
GO_REGULATION_OF_ENDOTHELIAL_CELL_MIGRATION	1.833	0.006	0.049
GO_FIBROBLAST_PROLIFERATION	1.801	0.006	0.056
GO_BLOOD_VESSEL_ENDOTHELIAL_CELL_MIGRATION	1.770	0.011	0.063

Gene sets with NOM *p*-val < 0.05 and FDR *q*-val < 0.25 were considered significant

*GO* Gene Ontology, *NES* normalized enrichment score, *NOM* nominal, *FDR* false discovery rate

**Table 6:** Gene sets enriched in the low risk phenotype via GO

Gene set name	NES	NOM *p*-val	FDR *q*-val
GO_T_CELL_CYTOKINE_PRODUCTION	−1.594	0.034	0.227
GO_T_CELL_ACTIVATION_INVOLVED_IN_IMMUNE_RESPONSE	−1.721	0.036	0.195
GO_PRODUCTION_OF_MOLECULAR_MEDIATOR_OF_IMMUNE_RESPONSE	−1.740	0.034	0.192
GO_CD4_POSITIVE_ALPHA_BETA_T_CELL_ACTIVATION	−1.747	0.028	0.188
GO_CD4_POSITIVE_ALPHA_BETA_T_CELL_LINEAGE_COMMITMENT	−1.754	0.019	0.183
GO_CD8_POSITIVE_ALPHA_BETA_T_CELL_ACTIVATION	− 1.798	0.019	0.160
GO_T_CELL_DIFFERENTIATION_INVOLVED_IN_IMMUNE_RESPONSE	−1.807	0.024	0.161
GO_POSITIVE_REGULATION_OF_PROTEIN_DEACETYLATION	−1.836	0.006	0.146
GO_POSITIVE_REGULATION_OF_SIGNAL_TRANSDUCTION_BY_P53_CLASS_MEDIATOR	−1.838	0.004	0.149
GO_MHC_PROTEIN_COMPLEX	−1.838	0.002	0.150
GO_POSITIVE_REGULATION_OF_INTERLEUKIN_12_PRODUCTION	−1.859	0.006	0.146
GO_FATTY_ACID_BETA_OXIDATION	−1.872	0.004	0.147
GO_T_CELL_SELECTION	−1.889	0.017	0.156
GO_IMMUNOGLOBULIN_PRODUCTION	−1.898	0.002	0.163
GO_CD4_POSITIVE_OR_CD8_POSITIVE_ALPHA_BETA_T_CELL_LINEAGE_COMMITMENT	−1.919	0.004	0.157
GO_T_CELL_RECEPTOR_SIGNALING_PATHWAY	−1.920	0.008	0.161
GO_FATTY_ACID_CATABOLIC_PROCESS	−1.936	0.004	0.168
GO_POSITIVE_T_CELL_SELECTION	−1.944	0.004	0.194
GO_T_CELL_RECEPTOR_COMPLEX	−1.972	0.002	0.260
GO_MHC_PROTEIN_BINDING	−1.994	0.006	0.369

Gene sets with NOM *p*-val < 0.05 and FDR *q*-val < 0.25 were considered significant

*GO* Gene Ontology, *NES* normalized enrichment score, *NOM* nominal, *FDR* false discovery rate

**Table 7:** Gene sets enriched in the high risk phenotype via KEGG

Gene set name	NES	NOM *p*-val	FDR *q*-val
KEGG_FOCAL_ADHESION	2.390	< 0.001	< 0.001
KEGG_ECM_RECEPTOR_INTERACTION	2.374	< 0.001	< 0.001
KEGG_HYPERTROPHIC_CARDIOMYOPATHY_HCM	2.115	0.002	0.003
KEGG_GAP_JUNCTION	1.932	< 0.001	0.027
KEGG_CALCIUM_SIGNALING_PATHWAY	1.896	0.002	0.031
KEGG_VASCULAR_SMOOTH_MUSCLE_CONTRACTION	1.860	0.006	0.035
KEGG_ADHERENS_JUNCTION	1.809	0.008	0.042
KEGG_MELANOGENESIS	1.763	< 0.001	0.057
KEGG_TGF_BETA_SIGNALING_PATHWAY	1.648	0.031	0.114
KEGG_PATHWAYS_IN_CANCER	1.633	0.017	0.118
KEGG_NEUROACTIVE_LIGAND_RECEPTOR_INTERACTION	1.625	0.017	0.119
KEGG_WNT_SIGNALING_PATHWAY	1.606	0.034	0.117
KEGG_MAPK_SIGNALING_PATHWAY	1.590	0.028	0.123
KEGG_GLYCOSAMINOGLYCAN_DEGRADATION	1.586	0.038	0.121
KEGG_PROGESTERONE_MEDIATED_OOCYTE_MATURATION	1.572	0.041	0.126
KEGG_HEDGEHOG_SIGNALING_PATHWAY	1.568	0.030	0.124
KEGG_GNRH_SIGNALING_PATHWAY	1.537	0.028	0.137
KEGG_TIGHT_JUNCTION	1.522	0.039	0.138

Gene sets with NOM *p*-val < 0.05 and FDR *q*-val < 0.25 were considered significant

*KEGG* The Kyoto Encyclopedia of Genes and Genomes, *NES* normalized enrichment score, *NOM* nominal, *FDR* false discovery rate

**Table 8:** Gene sets enriched in low risk phenotype via KEGG

Gene set name	NES	NOM *p*-val	FDR *q*-val
KEGG_PEROXISOME	−1.986	< 0.001	1.467
KEGG_LINOLEIC_ACID_METABOLISM	−1.934	< 0.001	1.397
KEGG_ANTIGEN_PROCESSING_AND_PRESENTATION	−2.150	0.002	1.568
KEGG_CYTOSOLIC_DNA_SENSING_PATHWAY	−2.070	0.004	1.419
KEGG_RIG_I_LIKE_RECEPTOR_SIGNALING_PATHWAY	−1.912	0.008	1.425
KEGG_AUTOIMMUNE_THYROID_DISEASE	−2.013	0.008	1.479
KEGG_GRAFT_VERSUS_HOST_DISEASE	−1.959	0.008	1.425
KEGG_ALLOGRAFT_REJECTION	−1.926	0.012	1.431
KEGG_PRIMARY_IMMUNODEFICIENCY	−1.777	0.028	1.053
KEGG_METABOLISM_OF_XENOBIOTICS_BY_CYTOCHROME_P450	−1.678	0.030	0.943
KEGG_ETHER_LIPID_METABOLISM	−1.507	0.031	0.691
KEGG_AMINOACYL_TRNA_BIOSYNTHESIS	−1.645	0.036	0.895
KEGG_INTESTINAL_IMMUNE_NETWORK_FOR_IGA_PRODUCTION	−1.716	0.039	0.969

Gene sets with NOM *p*-val < 0.05 and FDR *q*-val < 0.25 are considered as significant

*KEGG* The Kyoto Encyclopedia of Genes and Genomes, *NES* normalized enrichment score, *NOM* nominal, *FDR* false discovery rate

## Discussion

BC is the most common malignant tumor of the urinary system and has complex biological behavior, a high recurrence rate and a high metastasis rate [39]. Among the BC treatments being studied, immunotherapy seems to be the most promising [40]. In 1990, BCG was approved for immunotherapy for BC and achieved great success, but it should be recognized that approximately 40% of BC patients have no response to BCG, and even 15% of BCs progress to MIBC after treatment [41, 42]. In recent years, new findings have suggested that tumor cells can escape the immune response by affecting immune checkpoints [43, 44]. Therefore, research on ICIs to prevent immune escape is receiving much attention at present [45–47]. Five ICIs, pembrolizumab, nivolumab, atezolizumab, durvalumab and avelumab, have been approved by the Food and Drug Administration (FDA) for the treatment of advanced and metastatic BC [48]. One study indicated that in PD-L1-positive BC patients, durvalumab showed controlled safety and meaningful clinical activity [49]. In summary, the immunology of BC is worthy of further exploration. Importantly, in this study, we constructed a prognostic risk signature by using IRGPs, which is significant for furthering the understanding of the immune response in BC.

In general, GEPs identified from large public databases can be used to build risk signatures. However, there are many deficiencies in traditional construction schemes. The overfitting of a small sample training set and lack of sufficient verification can reduce the accuracy of statistics [50]. In some public databases, such as TCGA, the number of tumor samples is far greater than that of normal samples, and paired data are scarce [51]. If a model is constructed by screening differentially expressed genes (tumor vs normal), its robustness is doubtful. This problem can be solved by jointly considering GEPs from multiple databases. Unfortunately, the data from multiple platforms are difficult to standardize because of biological heterogeneity and technical biases [52]. Hence, we built our risk signature using IRGPs, which were identified based on the relative ranking and pairwise comparison of gene expression within the same patient, thus overcoming the batch effects encountered when data from different platforms are analyzed [25, 53]. Additionally, this new method also avoids issues related to an imbalance between the numbers of tumor samples and normal samples. Some tumor studies have shown convincing results using this method [54, 55].

Our risk signature was constructed with 30 IRGPs consisting of 28 IRGs. A high risk score independently predicted poor prognosis in BC patients. CD14 was among the 28 IRGs, and BC cells with high CD14 expression have been shown to produce tumor-promoting inflammation and promote tumor cell proliferation [56]. Joint blockade of complement C5a receptor 1 (C5AR1) and PD-1 prevented lung cancer metastasis and improved the prognosis of patients [57]. Overexpression of Dickkopf WNT signaling pathway inhibitor 1 (DKK1) has been shown to be related to poor OS in patients with BC [58]. An increased level of serum interleukin 18 (IL18) was found in patients with BC, which might be the result of the patients’ immune systems fighting to inhibit the growth of tumor cells [59]. Upregulated matrix metallopeptidase 9 (MMP9) is closely related to the metastasis of BC [60, 61]. In another study, the knockdown of pentraxin 3 (PTX3) activated the proliferation of BC cells and enhanced the metabolism of tumor cells [62]. In this study, CIBERSORT was used to evaluate immune cell infiltration in the different risk groups. The levels of memory B cells, plasma cells, M1 macrophages, CD8 T cells, activated memory CD4 T cells and follicular helper T cells were higher in the low-risk group than in the high-risk group. The levels of M0 macrophages, M2 macrophages, eosinophils and resting memory CD4 T cells were higher in the high-risk group than in the low-risk group. Recent studies have indicated that high infiltration levels of CD8 T cells and CD4 T cells can exert anti-BC effects [63, 64]. High levels of M2 macrophages were significantly associated with poor prognosis in patients with BC, and metastasis of BC cells was inhibited by inducing M1 macrophage polarization [65]. In the low-risk group, the main effector immune cell infiltration level was increased, implying a stronger immune response, which may be the reason for the better prognosis of the low-risk group.

The level of TMB and the expression of immune checkpoints (PD-1, CTLA4 and LAG3) were both significantly negatively correlated with the risk score, suggesting that in the low-risk group, with the immune response enhanced, the expression of immune checkpoints was also increased, but fortunately, the immune response was activated more than suppressed, and the response to immunotherapy or ICIs could be more effective.

ESTIMATE was performed to analyze the association between the TME and the risk score. The TME, the cellular environment of tumor cells, is mainly composed of immune cells, stromal cells, extracellular matrix (ECM), small organelles and secreted proteins [66]. The results of our research demonstrated that the infiltration level of stromal cells was upregulated in the high-risk group, but there was no correlation between the immune score, tumor purity and risk score. Due to many different subtypes of immune cells, although some infiltrating immune cells in the high-risk group could not produce immune effects, they were clustered in both the high-risk group and the low-risk group via CIBERSORT analysis, and it was possible that there was no difference in the total immune score between the two groups. Additionally, low immune scores and the level of TMB were associated with poor OS in patients with BC. Therefore, we believe that immunotherapy is effective for patients with BC.

We did the same analysis with the GSE31684 dataset. Unfortunately, the results from the TCGA data were only partially observed in the results of the analysis of the GSE31684 dataset. There might be many reasons for this discrepancy. First, the changes in the TME and immune checkpoints were quite complicated. Second, the sample size of each BC data cohort in the GEO database was smaller than that in the cohorts in the TCGA database, and no mutation data were included. Third, the sequencing methods and data normalization used for each data cohort in the GEO database were not as advanced and rigorous as those used for the TCGA data cohorts. Given the deficiencies of the GEO data, the IMvigor210 dataset was also used for analysis, and we observed that more immune-related changes were found in the TCGA data than in the IMvigor210 data, but some results were still inconsistent. This discrepancy may be because the included samples were all advanced metastatic BC. Despite the limitations of the validated cohorts, we still found that many immune-related changes were consistent across the three cohorts (TCGA, GEO and IMvigor210), some immune cells (including M0 macrophages, M1 macrophages, M2 macrophages, activated memory CD4 T cells and resting memory CD4 T cells) showed the same changes. Moreover, TMB and some immune checkpoints also showed the same changes in the IMvigor210 data (there are no mutation data in GSE31684), suggesting that immunotherapy can achieve significant benefits in low-risk BC patients.

Finally, the molecular mechanisms underlying the risk score were explored via GSEA. The GO results showed that gene sets related to the ECM, stromal cells (chondrocytes, endothelial cells and fibroblasts) and epithelial-mesenchymal transition (EMT) were enriched in the high-risk group. Focal adhesion and ECM-receptor interaction, all connected with ECM, were the top two significant enrichment pathways in the high-risk group via KEGG. The enrichment of stromal cells in the high-risk group was consistent with the ESTIMATE results. However, we think it was valuable to discover that ECM, which constitutes scaffolds of tissues and organs, was enriched in the high-risk group [67]. The ECM is one of the most abundant components in the TME, and as the key to maintaining tissue homeostasis, the ECM is a dynamic environment, and ECM disorder can promote tumor occurrence, progression, and metastasis by inducing EMT [68–72]. The literature has shown that focal adhesion-related molecules, such as focal adhesion kinases (FAKs), play a vital role in EMT and upregulate the metastatic capacity of tumor cells in BC [73–75]. We also found that a high risk score was related to advanced M stage (metastasis). In the low-risk group, we found that there were a number of immune-related pathways in the enriched pathways and biofunctions via GO and KEGG, such as T cell receptor complex, immunoglobulin production, CD4-positive or CD8-positive alpha-beta T cell lineage commitment, primary immunodeficiency, intestinal immune network for IgA production and RIG I-like receptor signaling pathway, which might imply the activation of immune responses in the low-risk group.

However, the limitations of our study should be acknowledged. First, our study was a retrospective analysis, and the results need to be verified by a prospective cohort study. Second, the specific mechanism of the immunological parameters changing with the risk score was not studied in depth. Third, our analysis of the biofunctions underlying the risk score was not verified by in vitro or in vivo experiments.

Although our research had some limitations, the IRGP risk signature that we constructed for BC could predict the prognosis of patients, and use of this signature will be helpful for individualized treatment decisions, clinical decision-making and evaluation of the benefits of immunotherapy. In addition, the relevant genes included in the risk signature could also be used for further research to identify new therapeutic targets for BC.

## Conclusions

In this study, we used a new tool, IRGPs, to build a risk signature to predict the prognosis of BC. By evaluating immune parameters and molecular mechanisms, we gained further understanding of the mechanisms underlying the risk signature. The signature could also be used as a tool to predict the effect of immunotherapy in patients with BC.

## Supplementary Information

**Additional file 1: Supplementary Fig. 1.** Subgroup analyses (GEO). Subgroup analyses were performed based on age (A-B), clinical stage (C-D) and T stage (E-F) to confirm the robustness of the risk signature. The median of age was 68.015. GEO, Gene Expression Omnibus.

**Additional file 2: Supplementary Fig. 2.** Subgroup analyses (IMvigor210). Subgroup analyses were performed based on age (A-B) and subtype (C-D) to confirm the robustness of the risk signature.

**Additional file 3: Supplementary Fig. 3.** Immunological parameters and risk scores (GEO). The expression levels of BTLA (A) and CTLA4 (B) were significantly negatively correlated with the risk score. However, there was no significant correlation between the risk score and other immune checkpoints, including PD-1 (C), PD-L1 (D), LAG3 (E), and HAVCR2 (F). The immune score (G) was significantly negatively correlated with the risk score. There was no significant correlation between the stromal score (H), tumor purity (I) and risk score. GEO, Gene Expression Omnibus; PD-1, programmed death-1; CTLA-4, cytotoxic T lymphocyte antigen-4; LAG3, lymphocyte activating 3; PD-L1, programmed death ligand-1; BTLA, B and T lymphocyte associated; HAVCR2, hepatitis A virus cellular receptor 2; BC, bladder cancer.

**Additional file 4: Supplementary Fig. 4.** Immunological parameters and risk scores (IMvigor210). The expression levels of PD-1 (A), BTLA (B), and CTLA4 (C) were significantly negatively correlated with the risk score. However, there was no significant correlation between the risk score and other immune checkpoints, including PD-L1 (D), LAG3 (E), and HAVCR2 (F). The stromal score (G) was significantly positively correlated with the risk score. The immune score (H) was significantly negatively correlated with the risk score. There was no significant correlation between tumor purity (I) and the risk score. PD-1, programmed death-1; CTLA-4, cytotoxic T lymphocyte antigen-4; LAG3, lymphocyte activating 3; PD-L1, programmed death ligand-1; BTLA, B and T lymphocyte associated; HAVCR2, hepatitis A virus cellular receptor 2; BC, bladder cancer.

## Data Availability

The datasets generated and/or analyzed during the current study are available in the TCGA repository (https://portal.gdc.cancer.gov/), GSE31684 repository, (https://www.ncbi.nlm.nih.gov/geo/query/acc.cgi?acc=GSE31684), and IMvigor210 (http://research-pub.gene.com/IMvigor210CoreBiologies/).

## Abbreviations

BC: Bladder cancer
NMIBC: Nonmuscle-invasive bladder cancer
MIBC: Muscle-invasive bladder cancer
BCG: Bacillus Calmette-Guerin
IL-2: Interleukin-2
IRGPs: Immune-related gene pairs
GSEA: Gene set enrichment analysis
ECM: Extracellular matrix
TURBT: Transurethral resection of bladder tumor
GMCSF: Granulocyte-macrophage colony-stimulating factor
ICIs: Immune checkpoint inhibitors
PD-1: Programmed death-1
PD-L1: Programmed death ligand-1
CTLA-4: Cytotoxic T lymphocyte antigen-4
TMB: Tumor mutation burden
TCGA: The Cancer Genome Atlas
LASSO: Least absolute shrinkage and selection operator
GEO: Gene Expression Omnibus
IRGs: Immune-related genes
TME: Tumor microenvironment
ImmPort: Immunology Database and Analysis Portal
ROC: Receiver operating characteristic
OS: Overall survival
CIBERSORT: Cell type identification by estimating relative subsets of RNA transcripts
GEPs: Gene expression profiles
LAG3: Lymphocyte activating 3
BTLA: B and T lymphocyte associated
HAVCR2: Hepatitis A virus cellular receptor 2
ESTIMATE: Estimation of Stromal and Immune cells in Malignant Tumor tissues using Expression data
NESs: Normalized enrichment scores
NOM *P* value: Nominal *P* value
FDR Q value: False discovery rate Q value
MSigDB: Molecular Signatures Database
AUC: Area under the curve
HR: Hazard ratio
GO: Gene Ontology
KEGG: Kyoto Encyclopedia of Genes and Genomes
C5AR1: C5a receptor 1
DKK1: Dickkopf WNT signaling pathway inhibitor 1
IL-18: Interleukin 18
MMP9: Matrix metallopeptidase 9
PTX3: Pentraxin 3
EMT: Epithelial-mesenchymal transition
FAKs: Focal adhesion kinases

## References

[1] SEER cancer statistics review, 1975–2016, National Cancer Institute. Bethesda. https://seer.cancer.gov/csr/based on November 2018 SEER data submission, posted to the SEER web site.

[2] Nabavizadeh R, Bobrek K, Master VA (2020). Risk stratification for bladder cancer: biomarkers of inflammation and immune activation. Urol Oncol.

[3] Li F, Guo H, Wang Y, Liu B, Zhou H (2020). Profiles of tumor-infiltrating immune cells and prognostic genes associated with the microenvironment of bladder cancer. Int Immunopharmacol.

[4] Rouanne M, Roumiguié M, Houédé N, Masson-Lecomte A, Colin P, Pignot G, Larré S, Xylinas E, Rouprêt M, Neuzillet Y (2018). Development of immunotherapy in bladder cancer: present and future on targeting PD(L)1 and CTLA-4 pathways. World J Urol.

[5] von der Maase H, Sengelov L, Roberts JT, Ricci S, Dogliotti L, Oliver T, Moore MJ, Zimmermann A, Arning M (2005). Long-term survival results of a randomized trial comparing gemcitabine plus cisplatin, with methotrexate, vinblastine, doxorubicin, plus cisplatin in patients with bladder cancer. J Clin Oncol.

[6] Duan S, Wang P, Liu F, Huang H, An W, Pan S, Wang X (2019). Novel immune-risk score of gastric cancer: a molecular prediction model combining the value of immune-risk status and chemosensitivity. Cancer Med.

[7] Angell H, Galon J (2013). From the immune contexture to the Immunoscore: the role of prognostic and predictive immune markers in cancer. Curr Opin Immunol.

[8] Gentles AJ, Newman AM, Liu CL, Bratman SV, Feng W, Kim D, Nair VS, Xu Y, Khuong A, Hoang CD, Diehn M, West RB, Plevritis SK, Alizadeh AA (2015). The prognostic landscape of genes and infiltrating immune cells across human cancers. Nat Med.

[9] Carter BW, Halpenny DF, Ginsberg MS, Papadimitrakopoulou VA, de Groot PM. Immunotherapy in Non-Small Cell Lung Cancer Treatment: Current Status and the Role of Imaging. Journal of thoracic imaging. 2017;32(5):300-12. 10.1097/RTI.0000000000000291. 10.1097/RTI.0000000000000291 28786858

[10] Lin P, Guo YN, Shi L, Li XJ, Yang H, He Y, et al. Development of a prognostic index based on an immunogenomic landscape analysis of papillary thyroid cancer. Aging (Albany NY). 2019;11(2):480-500. 10.18632/aging.101754. 10.18632/aging.101754 PMC6366981 30661062

[11] Arora S, Velichinskii R, Lesh RW, Ali U, Kubiak M, Bansal P, Borghaei H, Edelman MJ, Boumber Y (2019). Existing and emerging biomarkers for immune checkpoint immunotherapy in solid tumors. Adv Ther.

[12] Kresowik TP, Griffith TS (2009). Bacillus Calmette-Guerin immunotherapy for urothelial carcinoma of the bladder. Immunotherapy.

[13] Pettenati C, Ingersoll MA (2018). Mechanisms of BCG immunotherapy and its outlook for bladder cancer. Nat Rev Urol.

[14] Steinberg RL, Nepple KG, Velaer KN, Thomas LJ, O'Donnell MA (2017). Quadruple immunotherapy of Bacillus Calmette-Guérin, interferon, interleukin-2, and granulocyte-macrophage colony-stimulating factor as salvage therapy for non-muscle-invasive bladder cancer. Urol Oncol.

[15] Böhle A, Jocham D, Bock PR (2003). Intravesical bacillus Calmette-Guerin versus mitomycin C for superficial bladder cancer: a formal meta-analysis of comparative studies on recurrence and toxicity. J Urol.

[16] Lamm DL, van der Meijden PM, Morales A, Brosman SA, Catalona WJ, Herr HW, Soloway MS, Steg A, Debruyne FM (1992). Incidence and treatment of complications of bacillus Calmette-Guerin intravesical therapy in superficial bladder cancer. J Urol.

[17] Roufas C, Chasiotis D, Makris A, Efstathiades C, Dimopoulos C, Zaravinos A (2018). The expression and prognostic impact of immune cytolytic activity-related markers in human malignancies: a comprehensive meta-analysis. Front Oncol.

[18] Gust KM, Rebhan K, Resch I, Shariat SF, Necchi A (2020). Immune checkpoint inhibition in muscle-invasive and locally advanced bladder cancer. Curr Opin Urol.

[19] Zhang C, Li Z, Qi F, Hu X, Luo J (2019). Exploration of the relationships between tumor mutation burden with immune infiltrates in clear cell renal cell carcinoma. Ann Transl Med.

[20] Forschner A, Battke F, Hadaschik D, Schulze M, Weißgraeber S, Han CT, Kopp M, Frick M, Klumpp B, Tietze N, Amaral T, Martus P, Sinnberg T, Eigentler T, Keim U, Garbe C, Döcker D, Biskup S (2019). Tumor mutation burden and circulating tumor DNA in combined CTLA-4 and PD-1 antibody therapy in metastatic melanoma - results of a prospective biomarker study. J Immunother Cancer.

[21] Wang F, Wei XL, Wang FH, Xu N, Shen L, Dai GH, Yuan XL, Chen Y, Yang SJ, Shi JH, Hu XC, Lin XY, Zhang QY, Feng JF, Ba Y, Liu YP, Li W, Shu YQ, Jiang Y, Li Q, Wang JW, Wu H, Feng H, Yao S, Xu RH (2019). Safety, efficacy and tumor mutational burden as a biomarker of overall survival benefit in chemo-refractory gastric cancer treated with toripalimab, a PD-1 antibody in phase Ib/II clinical trial NCT02915432. Ann Oncol.

[22] Goodman AM, Kato S, Bazhenova L, Patel SP, Frampton GM, Miller V, Stephens PJ, Daniels GA, Kurzrock R (2017). Tumor mutational burden as an independent predictor of response to immunotherapy in diverse cancers. Mol Cancer Ther.

[23] Bhattacharya S, Andorf S, Gomes L, Dunn P, Schaefer H, Pontius J, et al. ImmPort: disseminating data to the public for the future of immunology. Immunologic research. 2014;58(2-3):234-9. 10.1007/s12026-014-8516-1. 10.1007/s12026-014-8516-1 24791905

[24] Kang K, Xie F, Mao J, Bai Y, Wang X (2020). Significance of tumor mutation burden in immune infiltration and prognosis in cutaneous melanoma. Front Oncol.

[25] Sun XY, Yu SZ, Zhang HP, Li J, Guo WZ, Zhang SJ (2020). A signature of 33 immune-related gene pairs predicts clinical outcome in hepatocellular carcinoma. Cancer Med.

[26] Luo J, Liu P, Wang L, Huang Y, Wang Y, Geng W, Chen D, Bai Y, Yang Z (2020). Establishment of an immune-related gene pair model to predict colon adenocarcinoma prognosis. BMC Cancer.

[27] Qiu H, Hu X, He C, Yu B, Li Y, Li J (2020). Identification and validation of an individualized prognostic signature of bladder cancer based on seven immune related genes. Front Genet.

[28] Zhu J, Wang H, Ma T, He Y, Shen M, Song W, Wang JJ, Shi JP, Wu MY, Liu C, Wang WJ, Huang YQ (2020). Identification of immune-related genes as prognostic factors in bladder cancer. Sci Rep.

[29] Wang J, Shen C, Dong D, Zhong X, Wang Y, Yang X (2021). Identification and verification of an immune-related lncRNA signature for predicting the prognosis of patients with bladder cancer. Int Immunopharmacol.

[30] Xing Q, Liu S, Jiang S, Li T, Wang Z, Wang Y (2020). Prognostic model of 10 immune-related genes and identification of small molecule drugs in bladder urothelial carcinoma (BLCA). Transl Androl Urol.

[31] Wu Y, Zhang L, He S, Guan B, He A, Yang K, Gong Y, Li X, Zhou L (2020). Identification of immune-related LncRNA for predicting prognosis and immunotherapeutic response in bladder cancer. Aging (Albany NY).

[32] Deng Y, Hong X, Yu C, Li H, Wang Q, Zhang Y, Wang T, Wang X (2021). Preclinical analysis of novel prognostic transcription factors and immune-related gene signatures for bladder cancer via TCGA-based bioinformatic analysis. Oncol Lett.

[33] Newman AM, Liu CL, Green MR, Gentles AJ, Feng W, Xu Y, Hoang CD, Diehn M, Alizadeh AA (2015). Robust enumeration of cell subsets from tissue expression profiles. Nat Methods.

[34] Ye L, Zhang T, Kang Z, Guo G, Sun Y, Lin K, Huang Q, Shi X, Ni Z, Ding N, Zhao KN, Chang W, Wang J, Lin F, Xue X (2019). Tumor-infiltrating immune cells act as a marker for prognosis in colorectal cancer. Front Immunol.

[35] Zhang L, Zhu P, Tong Y, Wang Y, Ma H, Xia X, Zhou Y, Zhang X, Gao F, Shu P (2019). An immune-related gene pairs signature predicts overall survival in serous ovarian carcinoma. Onco Targets Ther.

[36] Yoshihara K, Shahmoradgoli M, Martinez E, Vegesna R, Kim H, Torres-Garcia W, Trevino V, Shen H, Laird PW, Levine DA (2013). Inferring tumour purity and stromal and immune cell admixture from expression data. Nat Commun.

[37] Li B, Geng R, Wu Q, Yang Q, Sun S, Zhu S, Xu Z, Sun S (2020). Alterations in immune-related genes as potential marker of prognosis in breast cancer. Front Oncol.

[38] Kanehisa M, Goto S (2000). KEGG: Kyoto encyclopedia of genes and genomes. Nucleic Acids Res.

[39] Alfred Witjes J, Lebret T, Compérat EM, Cowan NC, De Santis M, Bruins HM, Hernández V, Espinós EL, Dunn J, Rouanne M (2017). Updated 2016 EAU guidelines on muscle-invasive and metastatic bladder cancer. Eur Urol.

[40] Wołącewicz M, Hrynkiewicz R, Grywalska E, Suchojad T, Leksowski T, Roliński J, Niedźwiedzka-Rystwej P (2020). Immunotherapy in bladder cancer: current methods and future perspectives. Cancers.

[41] Herr HW, Morales A (2008). History of bacillus Calmette-Guerin and bladder cancer: an immunotherapy success story. J Urol.

[42] Seidl C (2020). Targets for therapy of bladder cancer. Semin Nucl Med.

[43] Bellmunt J, Powles T, Vogelzang NJ (2017). A review on the evolution of PD-1/PD-L1 immunotherapy for bladder cancer: the future is now. Cancer Treat Rev.

[44] Katz H, Wassie E, Alsharedi M (2017). Checkpoint inhibitors: the new treatment paradigm for urothelial bladder cancer. Med Oncol.

[45] Cunha LL, Marcello MA, Rocha-Santos V, Ward LS. Immunotherapy against endocrine malignancies: immune checkpoint inhibitors lead the way. Endocr Relat Cancer. 2017;24(12):T261-81. 10.1530/ERC-17-0222. 10.1530/ERC-17-0222 28893836

[46] Popovic A, Jaffee EM, Zaidi N. Emerging strategies for combination checkpoint modulators in cancer immunotherapy. The Journal of clinical investigation. 2018;128(8):3209-18. 10.1172/jci120775 10.1172/JCI120775 PMC6063475 30067248

[47] Kobold S, Pantelyushin S, Rataj F, Vom Berg J. Rationale for Combining Bispecific T Cell Activating Antibodies With Checkpoint Blockade for Cancer Therapy. Front Oncol 2018;8:285-93. 10.3389/fonc.2018.00285. 10.3389/fonc.2018.00285 PMC6068270 30090763

[48] Alhalabi O, Rafei H, Shah A, Siefker-Radtke A, Campbell M, Gao J (2019). Targeting advanced urothelial carcinoma-developing strategies. Curr Opin Oncol.

[49] Massard C, Gordon MS, Sharma S, Rafii S, Wainberg ZA, Luke J, Curiel TJ, Colon-Otero G, Hamid O, Sanborn RE, O’Donnell PH, Drakaki A, Tan W, Kurland JF, Rebelatto MC, Jin X, Blake-Haskins JA, Gupta A, Segal NH (2016). Safety and efficacy of durvalumab (MEDI4736), an anti-programmed cell death Ligand-1 immune checkpoint inhibitor, in patients with advanced urothelial bladder cancer. J Clin Oncol.

[50] Li B, Cui Y, Diehn M, Li R (2017). Development and validation of an individualized immune prognostic signature in early-stage nonsquamous non-small cell lung cancer. JAMA Oncol.

[51] Ouyang W, Ren L, Liu G, Chi X, Wei H (2019). LncRNA MIR4435-2HG predicts poor prognosis in patients with colorectal cancer. PeerJ.

[52] Leek JT, Scharpf RB, Bravo HC, Simcha D, Langmead B, Johnson WE, Geman D, Baggerly K, Irizarry RA (2010). Tackling the widespread and critical impact of batch effects in high-throughput data. Nat Rev Genet.

[53] Eddy JA, Sung J, Geman D, Price ND (2010). Relative expression analysis for molecular cancer diagnosis and prognosis. Technol Cancer Res Treat.

[54] Popovici V, Budinska E, Tejpar S, Weinrich S, Estrella H, Hodgson G, Van Cutsem E, Xie T, Bosman FT, Roth AD (2012). Identification of a poor-prognosis BRAF-mutant-like population of patients with colon cancer. J Clin Oncol.

[55] Peng PL, Zhou XY, Yi GD, Chen PF, Wang F, Dong WG (2018). Identification of a novel gene pairs signature in the prognosis of gastric cancer. Cancer Med.

[56] Cheah MT, Chen JY, Sahoo D, Contreras-Trujillo H, Volkmer AK, Scheeren FA, Volkmer JP, Weissman IL (2015). CD14-expressing cancer cells establish the inflammatory and proliferative tumor microenvironment in bladder cancer. Proc Natl Acad Sci U S A.

[57] Ajona D, Ortiz-Espinosa S, Moreno H, Lozano T, Pajares MJ, Agorreta J, Bértolo C, Lasarte JJ, Vicent S, Hoehlig K, Vater A, Lecanda F, Montuenga LM, Pio R (2017). A combined PD-1/C5a blockade synergistically protects against lung cancer growth and metastasis. Cancer Discov.

[58] Wei R, Rodrìguez RA, Mullor M, Tan Z, Gui Y, Hu J, Zhu T, Huang X, Zhu Y, Xu J (2020). Analyzing the prognostic value of DKK1 expression in human cancers based on bioinformatics. Ann Transl Med.

[59] Bukan N, Sözen S, Coskun U, Sancak B, Günel N, Bozkirli I, Senocak C (2003). Serum interleukin-18 and nitric oxide activity in bladder carcinoma. Eur Cytokine Netw.

[60] Qin Z, Wang Y, Tang J, Zhang L, Li R, Xue J, Han P, Wang W, Qin C, Xing Q (2018). High LINC01605 expression predicts poor prognosis and promotes tumor progression via up-regulation of MMP9 in bladder cancer. Biosci Rep.

[61] Liu F, Zhang H, Xie F, Tao D, Xiao X, Huang C, Wang M, Gu C, Zhang X, Jiang G (2020). Hsa_circ_0001361 promotes bladder cancer invasion and metastasis through miR-491-5p/MMP9 axis. Oncogene.

[62] Matarazzo S, Melocchi L, Rezzola S, Grillo E, Maccarinelli F, Giacomini A, Turati M, Taranto S, Zammataro L, Cerasuolo M (2019). Long pentraxin-3 follows and modulates bladder cancer progression. Cancers.

[63] Oh DY, Kwek SS, Raju SS, Li T, McCarthy E, Chow E, Aran D, Ilano A, Pai CS, Rancan C (2020). Intratumoral CD4(+) T cells mediate anti-tumor cytotoxicity in human bladder cancer. Cell.

[64] Hartana CA, Ahlén Bergman E, Zirakzadeh AA, Krantz D, Winerdal ME, Winerdal M, Johansson M, Alamdari F, Jakubczyk T, Glise H, Riklund K, Sherif A, Winqvist O (2018). Urothelial bladder cancer may suppress perforin expression in CD8+ T cells by an ICAM-1/TGFβ2 mediated pathway. PLoS One.

[65] Liu J, Duan X (2017). PA-MSHA induces apoptosis and suppresses metastasis by tumor associated macrophages in bladder cancer cells. Cancer Cell Int.

[66] Li P, Cao J, Li J, Yao Z, Han D, Ying L, Wang Z, Tian J (2020). Identification of prognostic biomarkers associated with stromal cell infiltration in muscle-invasive bladder cancer by bioinformatics analyses. Cancer Med.

[67] Eble JA, Niland S (2019). The extracellular matrix in tumor progression and metastasis. Clin Exp Metastasis.

[68] Luo Y, Zeng G, Wu S (2019). Identification of microenvironment-related prognostic genes in bladder cancer based on gene expression profile. Front Genet.

[69] Shintani Y, Hollingsworth MA, Wheelock MJ, Johnson KR (2006). Collagen I promotes metastasis in pancreatic cancer by activating c-Jun NH(2)-terminal kinase 1 and up-regulating N-cadherin expression. Cancer Res.

[70] Torzilli PA, Bourne JW, Cigler T, Vincent CT (2012). A new paradigm for mechanobiological mechanisms in tumor metastasis. Semin Cancer Biol.

[71] Quail DF, Joyce JA (2013). Microenvironmental regulation of tumor progression and metastasis. Nat Med.

[72] Hynes RO (2009). The extracellular matrix: not just pretty fibrils. Science.

[73] Li D, Zhang Y, Zhang H, Zhan C, Li X, Ba T, Qiu Z, Lv G, Zou C, E F (2018). CADM2, as a new target of miR-10b, promotes tumor metastasis through FAK/AKT pathway in hepatocellular carcinoma. J Exp Clin Cancer Res.

[74] Yang TY, Wu ML, Chang CI, Liu CI, Cheng TC, Wu YJ (2018). Bornyl cis-4-hydroxycinnamate suppresses cell metastasis of melanoma through FAK/PI3K/Akt/mTOR and MAPK signaling pathways and inhibition of the epithelial-to-mesenchymal transition. Int J Mol Sci.

[75] Kong DB, Chen F, Sima N (2017). Focal adhesion kinases crucially regulate TGFβ-induced migration and invasion of bladder cancer cells via Src kinase and E-cadherin. Onco Targets Ther.

