# Supplementary figures and images for: A novel immune-related gene pair prognostic signature for predicting overall survival in bladder cancer
Source: BMC Cancer. 2021 Jul 15;21:810. doi: 10.1186/s12885-021-08486-0 (PMC8281685; doi:10.1186/s12885-021-08486-0)

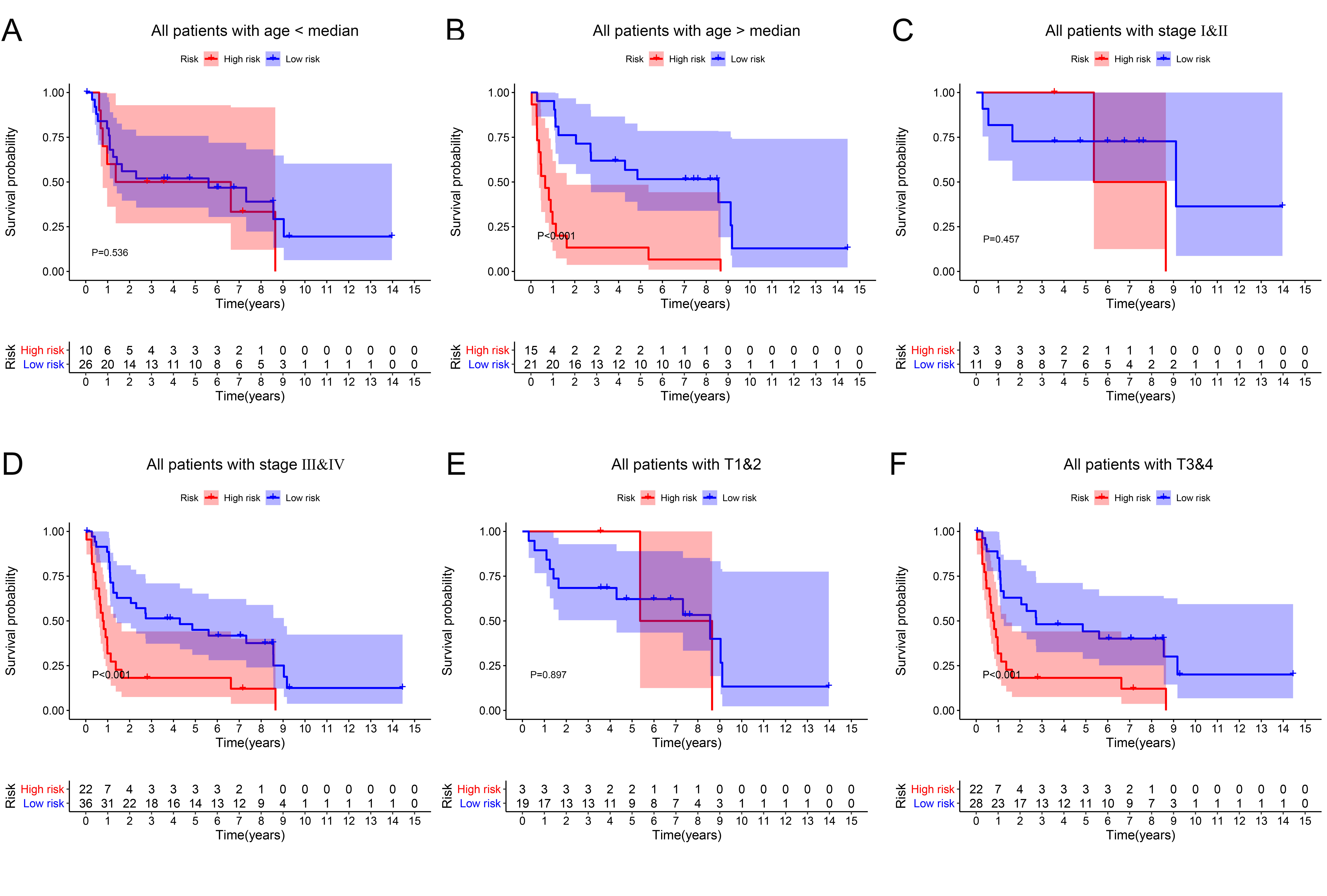

Supplement: Supplementary file 1 — Additional file 1: Supplementary Fig. 1. Subgroup analyses (GEO). Subgroup analyses were performed based on age (A-B), clinical stage (C-D) and T stage (E-F) to confirm the robustness of the risk signature. The median of age was 68.015. GEO, Gene Expression Omnibus. [file 12885_2021_8486_MOESM1_ESM.jpg]

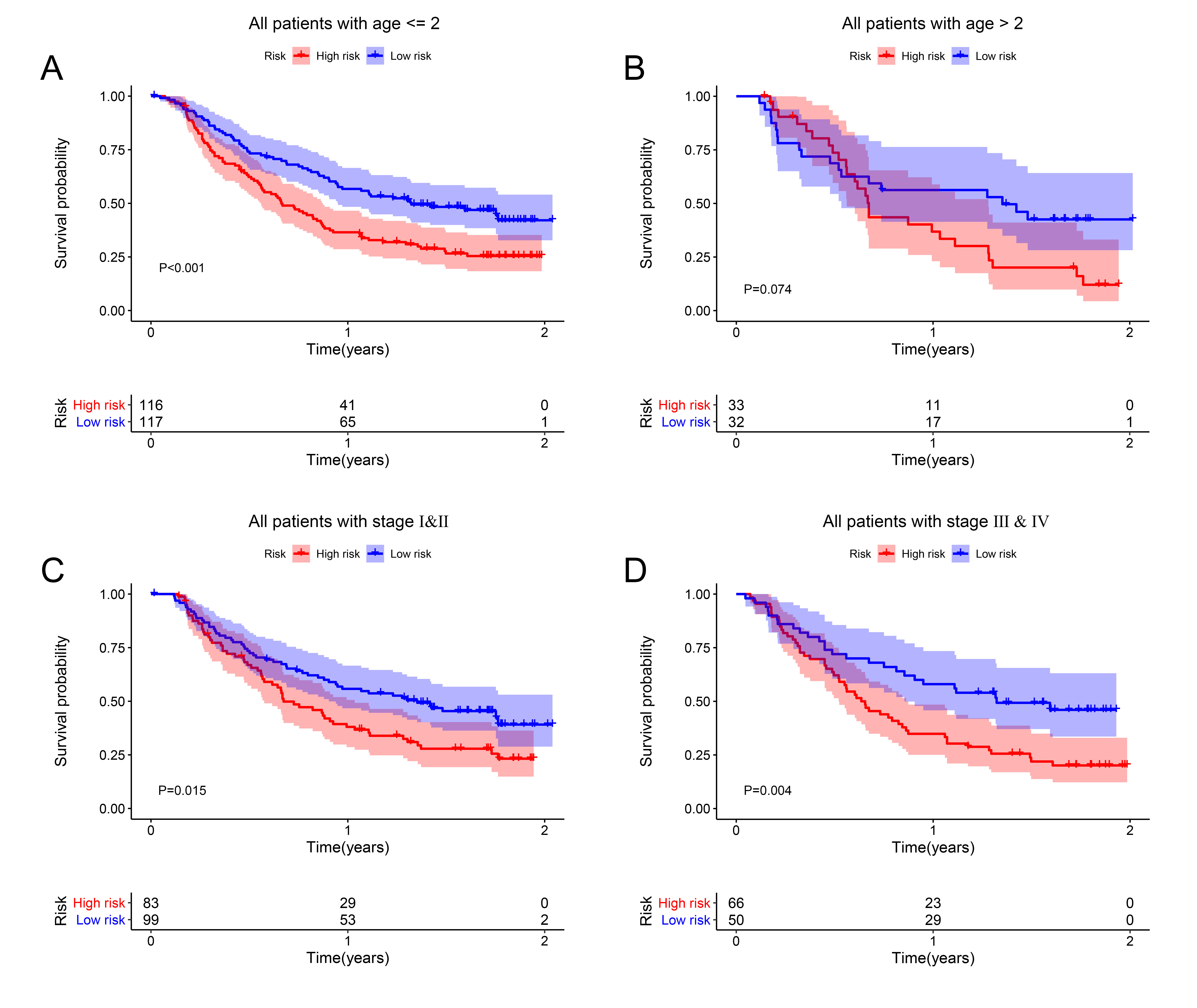

Supplement: Supplementary file 2 — Additional file 2: Supplementary Fig. 2. Subgroup analyses (IMvigor210). Subgroup analyses were performed based on age (A-B) and subtype (C-D) to confirm the robustness of the risk signature. [file 12885_2021_8486_MOESM2_ESM.jpg]
